# Targeting CRM1 for Progeria Syndrome Therapy

**DOI:** 10.1111/acel.14495

**Published:** 2025-01-27

**Authors:** Adriana Soto‐Ponce, Marlon De Ita, Susana Castro‐Obregón, Diego Cortez, Yosef Landesman, Jonathan J. Magaña, Susana Gonzalo, Tania Zavaleta, Angelica Soberano‐Nieto, Juan Unzueta, Isabel Arrieta‐Cruz, Porfirio Nava, Aurora Candelario‐Martínez, Ian García‐Aguirre, Bulmaro Cisneros

**Affiliations:** ^1^ Departamento de Genética y Biología Molecular Centro de Investigación y de Estudios Avanzados Ciudad de México Mexico; ^2^ Unidad de Investigación Médica en Genética Humana, Hospital de Pediatría, Centro Médico Nacional Siglo XXI, IMSSS Ciudad de México Mexico; ^3^ Instituto de Fisiología Celular, UNAM, Ciudad Universitaria Ciudad de México Mexico; ^4^ Centro de Ciencias Genómicas, UNAM Cuernavaca Mexico; ^5^ Karyopharm Therapeutics Newton Massachusetts USA; ^6^ Laboratorio de Medicina Genómica, Departamento de Genética (CENIAQ) Instituto Nacional de Rehabilitación‐Luis Guillermo Ibarra (INR‐LGII) Ciudad de México Mexico; ^7^ Departamento de Bioingeniería Escuela de Ingeniería y Ciencias, Tecnologico de Monterrey Ciudad de México Mexico; ^8^ Edward A. Doisy Department of Biochemistry and Molecular Biology Saint Louis University School of Medicine St. Louis Missouri USA; ^9^ Unidad Iztapalapa, División de Ciencias Biológicas y de la Salud Universidad Autónoma Metropolitana Ciudad de México Mexico; ^10^ Departamento de Investigación Básica, División de Investigación Instituto Nacional de Geriatría, Secretaría de Salud Ciudad de México Mexico; ^11^ Departamento de Fisiología, Biofísica y Neurociencias Centro de Investigación y de Estudios Avanzados Ciudad de México Mexico

**Keywords:** aging, progeria, senescence

## Abstract

Hutchinson‐Gilford progeria syndrome (HGPS) is a premature aging disease caused by progerin, a mutant variant of lamin A. Progerin anchors aberrantly to the nuclear envelope disrupting a plethora of cellular processes, which in turn elicits senescence. We previously showed that the chromosomal region maintenance 1 (CRM1)‐driven nuclear export pathway is abnormally enhanced in patient‐derived fibroblasts, due to overexpression of CRM1. Interestingly, pharmacological inhibition of CRM1 using leptomycin B rescues the senescent phenotype of HGPS fibroblasts, delineating CRM1 as a potential therapeutic target against HGPS. As a proof of concept, we analyzed the beneficial effects of pharmacologically modulating CRM1 in dermal fibroblasts from HGPS patients and the *LMNA^G609G/G609G^* mouse, using the first‐in‐class selective inhibitor of CRM1 termed selinexor. Remarkably, treatment of HGPS fibroblasts with selinexor mitigated senescence and promoted progerin clearance via autophagy, while at the transcriptional level restored the expression of numerous differentially‐expressed genes and rescued cellular processes linked to aging. In vivo, oral administration of selinexor to the progeric mouse resulted in decreased progerin immunostaining in the liver and aorta, decreased progerin levels in most liver, lung and kidney samples analyzed by immunoblotting, and improved aortic histopathology. Collectively our data indicate that selinexor exerts its geroprotective action by at least two mechanisms: normalizing the nucleocytoplasmic partition of proteins with a downstream effect on the aging‐associated transcriptome and decreasing progerin levels. Further investigation of the overall effect of selinexor on *Lmna^G609G/G609G^* mouse physiology, with emphasis in cardiovascular function is warranted, to determine its therapeutic utility for HGPS and aging‐associated disorders characterized by CRM1 overactivity.

## Graphical abstract

Pharmacological inhibition of CRM1 mediated by selinexor, the first‐in‐class selective inhibitor of CRM1, mitigates the senescent phenotype of Hutchinson‐Gilford progeria syndrome (HGPS) patients‐derived primary fibroblasts. Treatment of HGPS fibroblasts with selinexor promotes the clearances of progerin via autophagy activation, restores the expression of numerous differentially expressed genes, and rescues various aging‐linked cellular processes. Oral administration of selinexor to the LmnaG609G/G609G progeric mouse decreased progerin immunostaining in the liver and aorta and improved aortic histopathology.

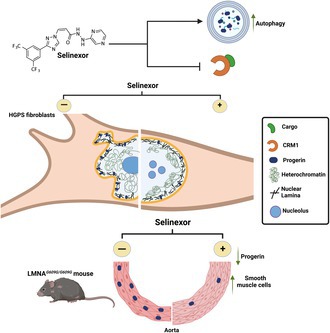

## 1 Introduction

In eukaryote cells, the intracellular traffic of proteins is tightly regulated to target to different subcellular compartments and attain critical concentration in each location in response to cellular requirements or specific stimuli, allowing cellular processes to occur in an organized manner and even simultaneously (Mor, White, and Fontoura 2014; Stewart 2022). The selective nucleocytoplasmic trafficking of proteins is operated by nuclear transporters, importins, and exportins, which recognize nuclear localization signal (NLS) and nuclear export signal (NES) motifs respectively, to drive the transit of protein cargoes in or out of the nucleus through the nuclear pore complex (NPC) (Mor, White, and Fontoura 2014; Stewart 2022). Proper cycles of import and export regulate a series of critical processes preserving thereby cellular homeostasis; indeed, nuclear transport dysfunction is a hallmark in various diseases, including neurodegeneration (Girdhar and Guo 2022), different types of cancer (Stelma et al. 2016), age‐related diseases and premature aging (Kumar and Lapierre 2021; Martins et al. 2020; Park et al. 2021).

Hutchinson‐Gilford progeria syndrome (HGPS) is a devasting premature aging disorder caused by a spontaneous single‐nucleotide substitution (1824 C > T) within the *LMNA* gene. Although this mutation is silent, it causes the usage of a cryptic donor splicing site in exon 11, which consequently deletes 150 base pairs in the prelamin A mRNA, and 50 amino acids in the prelamin A C‐terminus as a result. The lack of this stretch of amino acids impairs prelamin A maturation; typically, the C‐terminal CaaX motif undergoes farnesylation, endoproteolytic trimming of the last three residues, and methylation of the carboxyl‐terminal farnesyl‐cysteine, to facilitate its transport from the endoplasmic reticulum to the inner nuclear membrane. Finally, the last 15 amino acids of the protein, including the farnesylcysteine residue are clipped off by zinc metalloprotease ZMPSTE24 to render mature lamin A (De Sandre‐Giovannoli et al. 2003; Eriksson et al. 2003). In contrast, the mutant version of lamin A termed progerin, cannot undergo the second cleavage step due to the lack of the Zmptse24 cleavage site, thus remaining permanently farnesylated. Progerin stably attaches to the inner nuclear membrane exerting aberrant interactions with nuclear envelope (NE) proteins (including lamin A) and other nuclear compounds, which ultimately impairs a plethora of cellular processes, such as nuclear structure, genome stability, heterochromatin organization, telomere shortening, cell cycle regulation, transcriptional regulation, DNA damage response, and nucleocytoplasmic trafficking of proteins (De Sandre‐Giovannoli et al. 2003; Gonzalo, Kreienkamp, and Askjaer 2017).

Progerin causes nuclear membrane ruptures and blebs (Kim et al. 2024), as well as clustering of NPC (Robijns et al. 2018), which inevitably results in defective transport of molecules between the nucleus and the cytoplasm. Specifically, nuclear transport of E2‐conjugating enzyme Ubc9 and translocated promoter region (nucleoporin TPR), two high molecular weight proteins, is impaired in HGPS fibroblasts, due to an alteration in the gradient of Ran‐GTPase (Dworak et al. 2019; Kelley et al. 2011; Snow et al. 2013), which is required for the assembly of nuclear translocation complexes. Likewise, the non‐classic nuclear import mechanism driven by Transportin‐1 (TNPO‐1) is defective in HGPS cells, because TNPO‐1 is hijacked by the microtubule network precluding the import of nucleoporin Nup 153 (Larrieu et al. 2018). Concerning the opposite mechanism, we recently revealed that the nuclear export pathway mediated by chromosomal region maintenance 1 (CRM1), also known as XPO1, is exacerbated in HGPS human fibroblasts, which leads to an imbalance in the nucleocytoplasmic distribution of proteins (García‐Aguirre et al. 2019). CRM1 is the major nuclear export factor responsible for the export of several hundred protein and RNA substrates (Ishizawa et al. 2015). Of note, pharmacological inhibition of CRM1 using LMB rescued the senescence phenotype of HGPS cells (García‐Aguirre et al. 2019), highlighting the pivotal role of the nuclear export mechanisms in HGPS pathogenesis and pointing at CRM1 as a potential target for therapeutic intervention.

In this study, we set out to prove whether targeting the nuclear export pathway through pharmacological modulation of CRM1 would have a therapeutic impact on HGPS. Accordingly, we treated dermal fibroblasts derived from HGPS patients and the *LMNA* ^G609G/G609G^ mouse with the first‐ in‐ class selective inhibitor of CRM1 termed selinexor (Landes et al. 2023), and investigated the beneficial effects of this therapeutic agent at cellular and organismal levels.

## 2 Results

### 2.1 Selinexor Shows Low Toxicity and Effectively Inhibits CRM1 Activity in HGPS Fibroblasts

**FIGURE 1:**
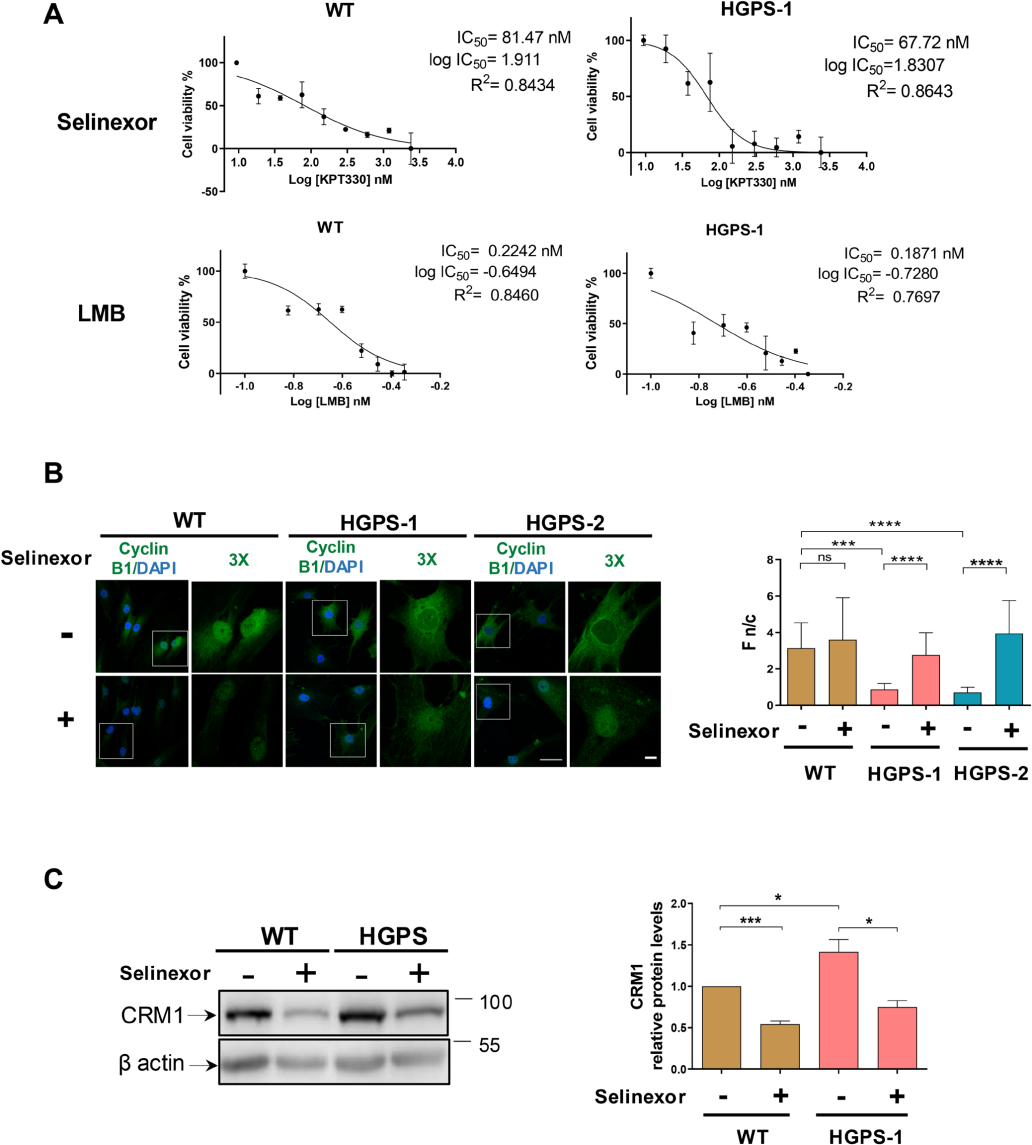
Effect of selinexor on cell viability and exportin CRM1 activity/levels in HGPS fibroblasts. (A) WT and HGPS‐1 fibroblasts seeded on 96‐well tissue culture plates were treated for 6 days with the indicated concentrations of selinexor and LMB, and then, the viability of cells was measured by MTT assays to calculate the IC_50_ of each compound. (B) WT and HGPS‐1/−2 cells grown on coverslips were treated for 24 with 60 nM selinexor or the vehicle alone, prior to be immunostained with cyclin B1 antibodies, and counterstaining with DAPI to visualize nuclei. Representative CLSM images are shown; bar scale, 10 μM. *Right*. The nuclear to cytoplasmic ratio of fluorescence (F n/c) ratio of cyclin B1 was calculated from three independent experiments using ImageJ software (*n* = 300 cells per experimental condition), and significant differences were determined by Mann–Whitney *U* test (****p < 0.0001; ***p < 0.001; ns, no significance). (C) Lysates from WT and HGPS‐1 fibroblasts previously treated for 6 days with 60 nM selinexor, or the vehicle alone were analyzed by western blotting using antibodies against CRM1 and actin (loading control). *Right*. The relative protein levels of CRM1 were calculated from three separate assays, and significant differences were obtained by unpaired *t*‐test (***p < 0.001; *p < 0.05).

To evaluate the ability of selinexor to mitigate the senescent marks of HGPS fibroblasts, an effective and safe dose of the compound was set up by calculating its 50% inhibitory concentration (IC_50_). The IC_50_ of LMB was also determined for comparison. To this end, the viability of WT and HGPS‐1 cells, previously treated for 6 days with varying doses of selinexor and LMB, was assessed by MTT assays. IC_50_ values of 81.47 and 67.72 nM were determined for selinexor in WT and HGPS‐1 cells respectively, while LMB showed IC_50_ values of 0.224 and 0.187 nM in WT and HGPS‐1 cells, respectively (Figure 1A). These results showed that HGPS‐1 cells had a slightly higher sensitivity than WT cells to both compounds; however, HGPS‐1 cells exhibited significantly lower sensitivity to selinexor than to LMB. Based on these results, we fixed a work concentration below the IC_50_ for selinexor (60 nM) and LMB (0.15 nM) for further experiments. Next, to examine the inhibitory effect of selinexor and LMB on CRM1 activity, the subcellular distribution of cyclin B1, a protein client of CRM1, was analyzed. The mislocalization of cyclin B1 from the nucleus to the cytoplasm was observed in HGPS‐1/‐2 fibroblasts, compared with WT cells (Figure 1B), which is causally linked to the overexpression of CRM1, as previously shown (García‐Aguirre et al. 2019). Nuclear accumulation of cyclin B1 was recovered upon treatment of HGPS‐1 cells with selinexor (Figure 1B) or LMB (Figure S1A), with quantitative analysis of the cyclin B1 nuclear/cytoplasmic fluorescence ratio (F N/C) validating these observations (Figure 1B, right graph and Figure S1A, right graph). Thus, an < IC_50_ concentration of selinexor was sufficient to block the nuclear export of this NES‐containing protein. Next, we sought to determine whether selinexor‐treated HGPS cells evoke the decrease of exportin CRM1, as previously reported in cancer cells (Gravina et al. 2015). A significant decline in the content of CRM1 was indeed found in WT and HGPS‐1 fibroblasts incubated with selinexor (Figure 1C). In contrast, the protein level of CRM1 was refractory to LMB treatment in both cell cultures (Figure S1B and the right graph). Finally, since selinexor acts as an apoptotic inducer in cancer cells (Gravina et al. 2015), it was necessary to rule out that selinexor has such an effect on HGPS fibroblasts. HGPS‐1 cells were treated with selinexor for 6 days before being analyzed for apoptosis using annexin V assays. No significant apoptosis was observed in selinexor‐treated HGPS‐1 cell cultures, while incubation with the apoptosis inducer staurosporine resulted in 50% of apoptotic cells in WT cultures (positive control), as shown by cytometry flux analyses (Figure S2A–C). In summary, selinexor could downregulate the nuclear protein export mechanism in HGPS cells by both pharmacologically inhibiting CRM1 activity and inducing CRM1 clearance, with tolerable effects on cell viability.

### 2.2 Selinexor‐Mediated Pharmacological Inhibition of CRM1 Rescues the Senescent Phenotype of HGPS Cells

**FIGURE 2:**
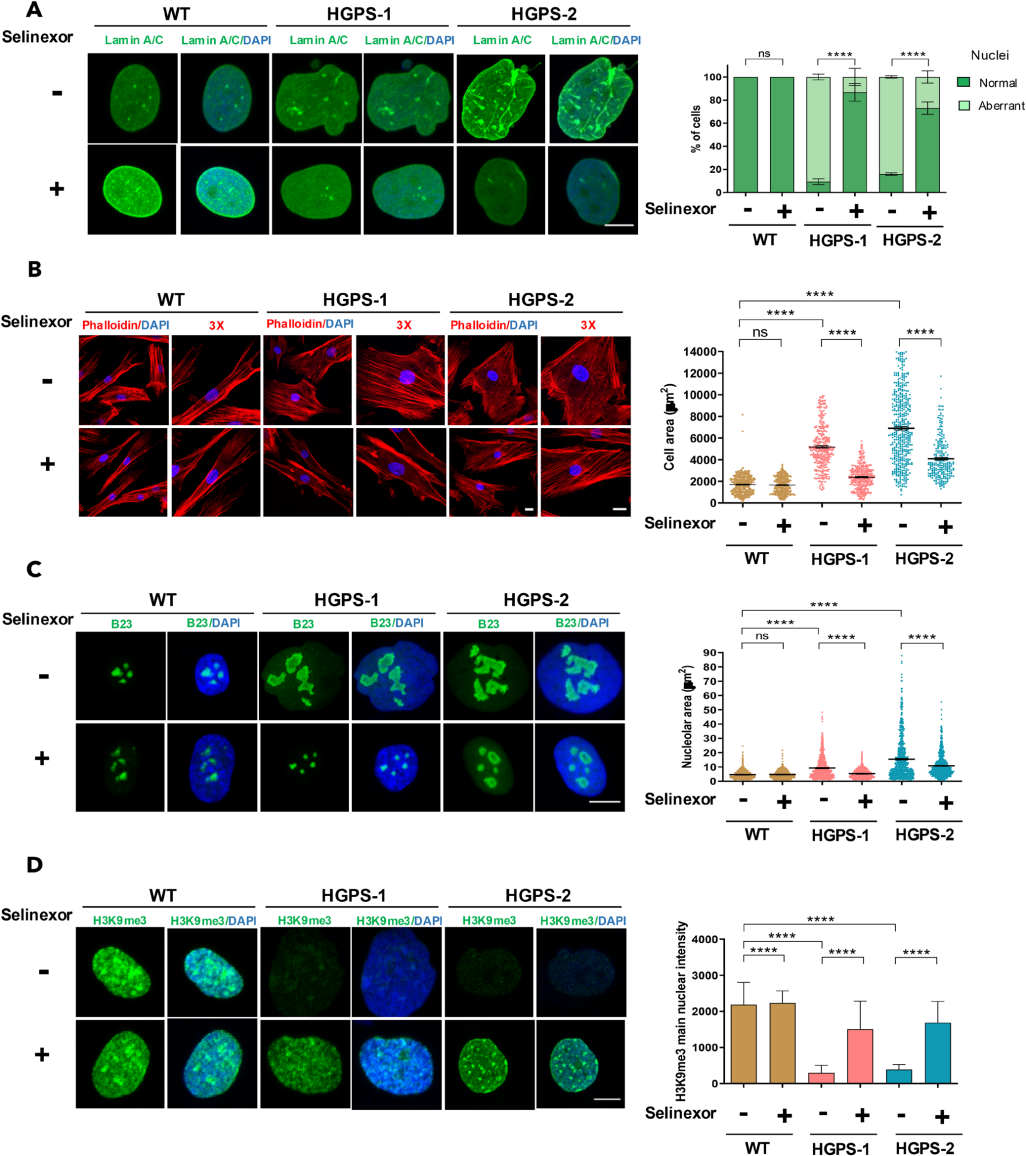
Treatment of HGPS cells with selinexor ameliorates their senescent features. WT and HGPS‐1/2 cells cultured on coverslips were treated for 6 days with 60 nM selinexor or the vehicle alone. (A) Cell preparations were immunolabeled for lamin A/C to analyze nuclear shape (A), stained with phalloidin to decorate actin‐based cytoskeleton and evaluate then cell morphology (B), immunolabeled for B23 (nucleolar protein marker) to analyze nucleolar morphology (C), or immunostained with antibodies against H3K9m3 to visualize heterochromatin (D). Cells were counterstained with DAPI to visualize nuclei prior to CLSM analysis, and typical images from three separate experiments are shown: Bar scale, 10 μM. *Right graphs*. The percentage of normal and irregularly shaped nuclei (A), the cellular (B) and nucleolar area (C), and the H3k9m3 fluorescence intensity (D) were scored (*n* = 300 cells per experimental condition), with significant differences determined by Mann–Whitney *U* test (****p < 0.0001; ns, no significance).

To assess the therapeutic potential of selinexor in vitro, different hallmarks of cellular senescence were analyzed in HGPS‐1/‐2 cells. Firstly, the impact of selinexor on nuclear morphology was evaluated by immunolabeling for lamin A/C; abnormally‐shaped nuclei, including blebbed, fissured, kidney‐shaped, and multi‐lobed nuclei, were observed in most vehicle‐treated HGPS‐1 (95%) and HGPS‐2 (90%) cell cultures (Figure 2A and right graph). By contrast, nuclei with oval shapes, like those of WT cells, were consistently found in HGPS‐1 (90%) and HGPS‐2 (73%) cell cultures upon selinexor treatment (Figure 2A and right graph). Morphometric analysis of nuclei using nuclear area and nuclear roundness parameters validated the improvement in nuclear morphology of HGPS‐1/‐2 cells in response to selinexor (Figure S3A). Next, the impact of selinexor on the senescent cellular morphology was analyzed: HGPS‐1 and HGPS‐2 fibroblasts were stained with phalloidin to decorate the actin‐based cytoskeleton and measure the cellular area. As a result of the treatment with selinexor, the typical flattened and enlarged shape of HGPS‐1/‐2 fibroblasts was shifted to the characteristic fusiform morphology of normal fibroblasts, (Figure 2B). Measuring of the cellular area confirmed a significant decrease in the size of selinexor‐treated HGPS‐1/HGPS‐2 fibroblasts, compared with their vehicle‐treated counterpart (right graph). Selinexor was also effective in preventing the formation of enlarged nucleoli, a well‐recognized feature of senescent cells (Buchwalter and Hetzer 2017; Phan, Khalid, and Iben 2019). A single nucleolus or two prominent nucleoli were consistently found in HGPS‐1/‐2 cells, while the treatment with selinexor resulted in both smaller nucleoli and an increased number of nucleoli per cell, as shown by immunofluorescence analysis for the nucleolar protein B23 (Figure 2C), and further assessing of nucleolar area (right graph). In addition, a substantial recovery of peripheral heterochromatin was observed in both HGPS cell cultures after selinexor treatment, as evidenced by analyzing and quantifying the heterochromatin marker H3K9me3 immunostaining (Figure 2D and right graph). On the other hand, treatment of HGPS cells with a < IC_50_ dose of LMB (0.15 nM) failed to alleviate any of the senescent characteristics analyzed; nonetheless, treatment with a > IC_50_ LMB dose (50 nM) was effective in ameliorating the senescent marks of HGPS cells (Figures S3B and S4A–D), as previously (García‐Aguirre et al. 2019).

**FIGURE 3:**
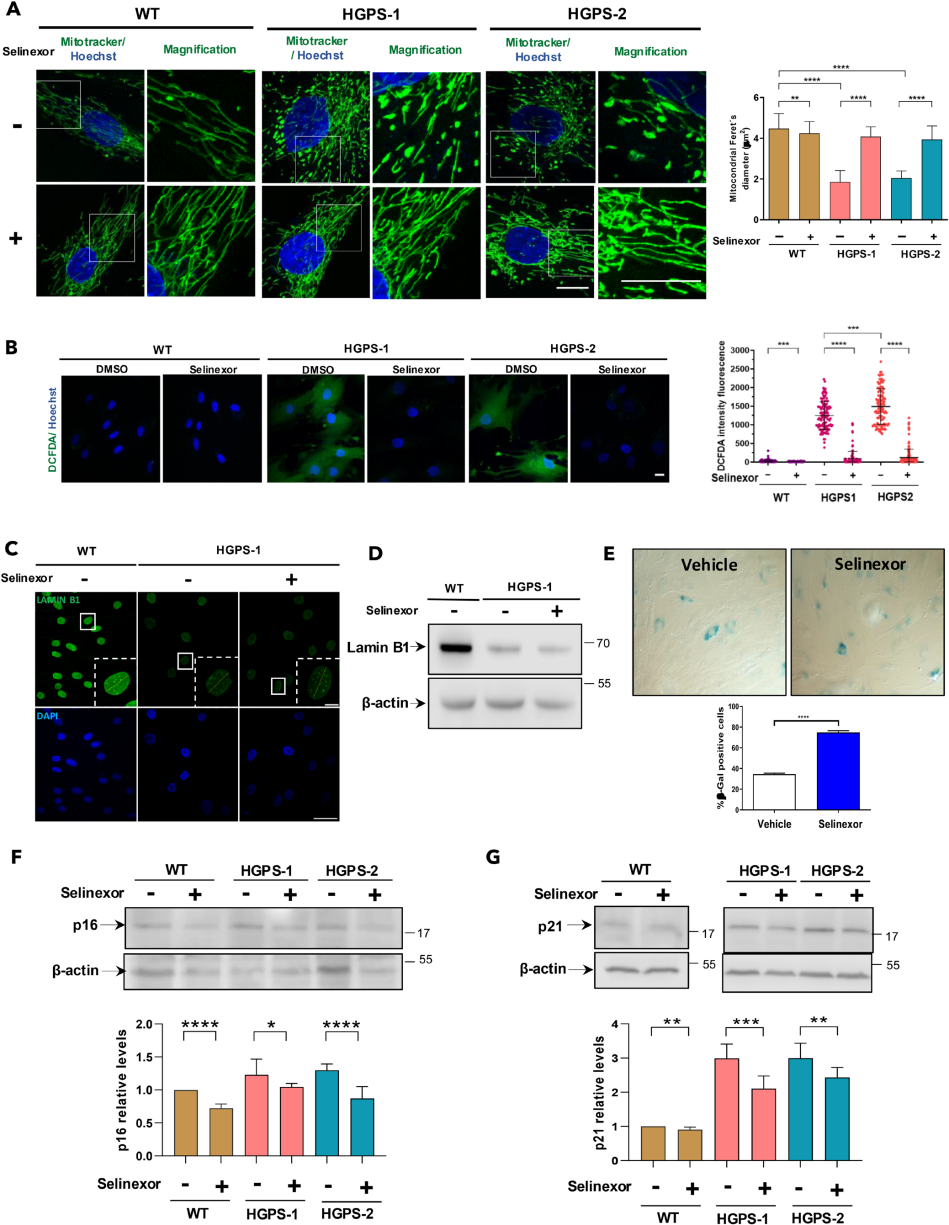
Selinexor prevented the entry of HGPS cells into senescence. (A) WT and HGPS‐1/2 fibroblasts grown on coverslips were treated with 60 nM selinexor or vehicle alone for 6 days and then subjected to MitoTracker Green staining. Nuclei were labeled with Hoechst prior to analysis by confocal microscopy. Representative images of labeled mitochondria are shown. Scale bar, 10 μm. *Right*. A morphometric analysis of mitochondria was conducted to calculate the diameter of the Ferret. The results correspond to the mean ± SD from three separate experiments with significant differences determined by an unpaired *t*‐test (*n* = 50 cells per experimental group; ****p < 0.0001). (B) WT and HGPS‐1/2 cells were incubated with 60 nM selinexor or the vehicle alone, prior to being incubated with H2DCFDA to detect ROS. Scale bar, 10 μm *Right*. The variability in H2DCFDA fluorescence intensity among the experimental groups was quantified using ImageJ (*n* = 130 cells per experimental condition), and statistically significant differences were identified through the nonparametric Mann–Whitney test (***pp < 0.001; ****p < 0.0001). (C) WT and HGPS‐1/2 fibroblasts cultured on coverslips were treated with 50 nM selinexor or vehicle alone for 6 days prior to immunofluorescence staining for lamin B1. Typical images are shown; scale bar, 10 μm. (D) Western blot analysis was conducted on lysate samples from WT and HGPS‐1/2 cell cultures previously treated for 6 days with 60 nM selinexor or the vehicle. Antibodies specific for lamin B1 and β‐actin (loading control) were used. A representative blot of two independent experiments is shown. (E) The activity of SA‐β‐gal was assessed (see Methods for details) and representative images from three separate experiments were acquired by light‐field microscopy. *Bottom*. The percentage of SA‐β‐gal positive cells was quantified (*n* = 300 cells per experimental condition) using NIS elements software (Nikon). (F and G) Lysates from WT and HGPS‐1/2 cell cultures previously treated with selinexor or the vehicle alone as above, were analyzed by western blotting using specific antibodies against p16 (F), p21^WAF1/CIP1^ and actin (loading control). *Down*. The relative levels of p21 and p16 were estimated from three separate experiments, with significant differences determined by unpaired *t*‐test (*p < 0.1; **p < 0.01; ***p < 0.001; ****p < 0.0001). The levels of p16 and p21^WAF1/CIP1^ from untreated WT cells were set at 1 for comparison.

Mitochondrial dysfunction is another relevant mark of senescent HGPS cells (Mateos et al. 2015; Monterrubio‐Ledezma et al. 2023); thus, we analyzed the effect of selinexor on mitochondrial morphology. WT and HGPS fibroblasts were incubated with MitoTracker Green dye, which stains mitochondria in living cells irrespective of the mitochondrial membrane potential. Most WT fibroblasts exhibited mitochondrial reticular networks comprising long tubules, whereas HGPS‐1/2 fibroblasts displayed a considerable number of short intermediate mitochondrial tubules, and fragmented/swollen mitochondria, which correspond to damaged mitochondria (Figure 3A). Remarkably, the majority of HGPS‐1 and HGPS‐2 fibroblasts treated with selinexor exhibited mitochondrial reticular networks that resemble those found in WT fibroblasts (Figure 3A). The quantification of mitochondrial morphology using Feret's diameter corroborated these findings (right graph). Mitochondrial dysfunction is associated with increased production of reactive species in senescent cells (Hajam et al. 2022); therefore, we sought to determine whether treatment with selinexor could ameliorate this alteration. The production of intracellular ROS was measured using the H₂DCFDA‐based assay (see Methods). The ROS‐dependent fluorescence was markedly elevated in HGPS‐1/2 cells relative to WT cells (Figure 3B). Conversely, the intensity of H2DCFDA fluorescence was undetectable in selinexor‐treated HGPS‐1/HGPS‐2 cells (Figure 3B), a finding corroborated by quantitative analysis (right panel). Given that decreased lamin B1 expression is a determinant of cellular senescence (Freund et al. 2012; Shimi et al. 2011), we sought to investigate whether treatment with selinexor could lead to the recovery of lamin B1 protein levels. However, no apparent recovery of lamin B1 was observed in selinexor‐treated HGPS cells by IF and WB assays (Figure 3C,D). Finally, we examined whether the senescence‐associated β‐galactosidase (SA‐β‐gal) activity that distinguishes senescent cells is reduced by selinexor. Unexpectedly, an increased percentage of HGPS‐1 fibroblasts with high senescence‐associated β‐galactosidase (SA‐β‐gal) activity was observed after the treatment with selinexor (Figure 3E).

As senescent cells cease to divide and remain irreversible arrested at G1 to avoid duplication of damaged genomes (Kumari and Jat 2021), we sought to determine whether alleviation of the senescent phenotype of HGPS cells mediated by selinexor is reflected on their cell cycle profile. As expected, most HGPS‐1/HGPS‐2 cells were found to be arrested at G0/G1 (~90%), with small pools of cells (5%–10%) at the S and M phases (Figure S2D). Interestingly, an evident reduction in the percentage of HGPS‐1/HGPS‐2 cells at G1 (25%–30%) with the concomitant increase of cells in S (10%–30%) and M (10%–15%) phases were obtained upon treatment with selinexor, as revealed by flow cytometry analyses (Figure S2D). To confirm the effect of selinexor on cell cycle progression at the molecular level, the cyclin‐dependent kinase inhibitors p16^INK4A^ and p21^WAF1/CIP1^ were analyzed. Increased levels of the senescent‐associated proteins p16 ^INK4A^ and p21^WAF1/CIP1^ were shown by HGPS‐1/HGPS‐2 cells, and further exposure of these cell cultures to selinexor resulted in a decline in the levels of both proteins (Figure 3F,G). Collectively, our results imply that normalization of CRM1 activity by selinexor treatment prevented the entry of HGPS fibroblasts into cellular senescence and consequently improved their physiology.

### 2.3 Selinexor Treatment Induces Progerin Clearance in HGPS Cells via Autophagy Activation

**FIGURE 4:**
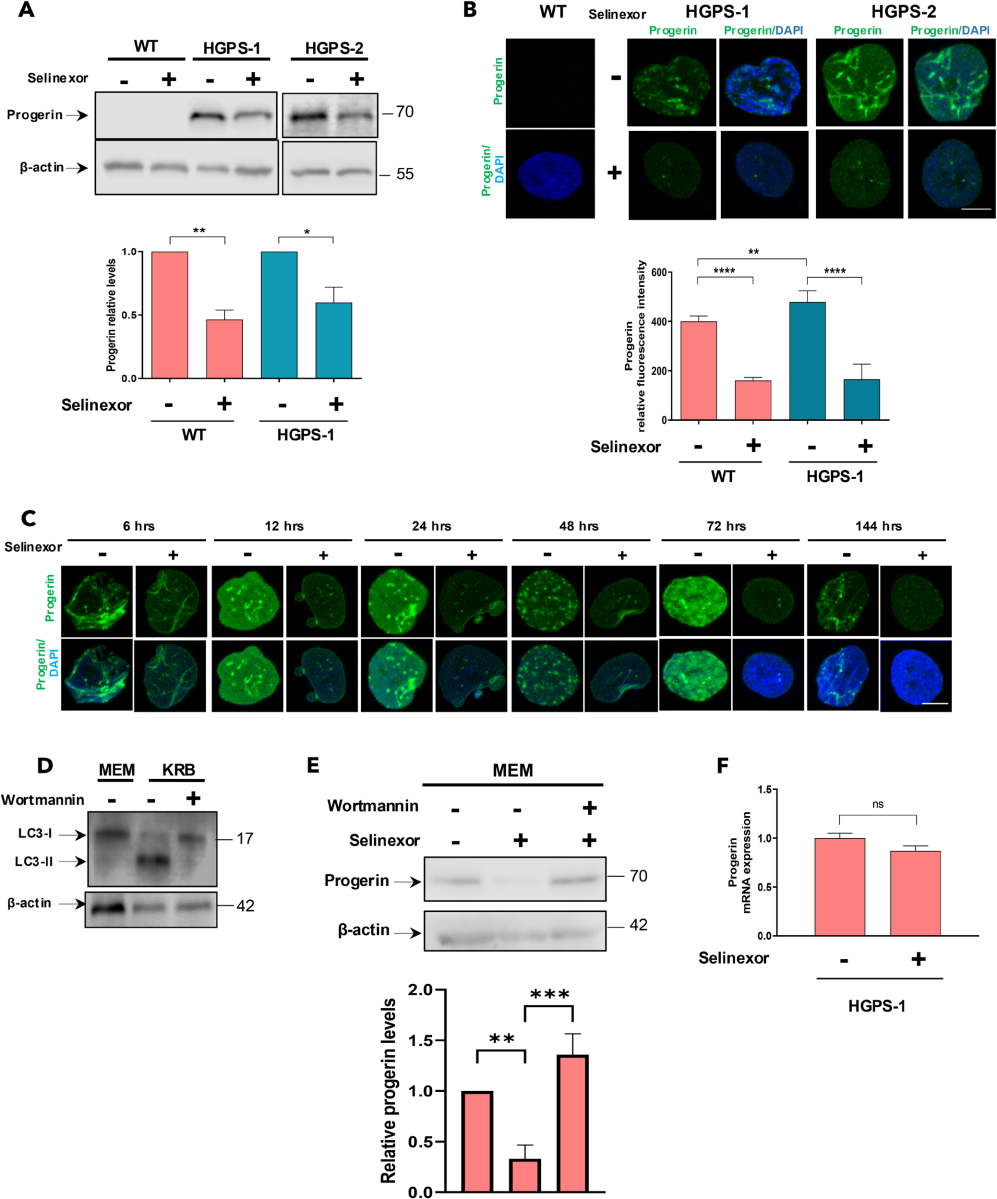
Selinexor treatment promotes progerin clearance in HGPS‐1/2 fibroblasts. (A) Lysates from WT and HGPS‐1/2 cells treated for 3 days with 60 nM selinexor, or the vehicle alone were isolated and further analyzed by western blotting using antibodies against progerin and actin (loading control). *Bottom*. Relative progerin levels from three separate experiments are shown, with statistical differences obtained by unpaired *t*‐test (*p < 0.1; **p < 0.01). (B) WT and HGPS‐1/2 fibroblasts seeded on coverslips were treated for 3 days with 60 nM selinexor or the vehicle alone, prior to be immunolabeled for progerin and counterstaining with DAPI to visualize nuclei. Typical images from three separate experiments are shown. Bar scale, 10 μM. The main nuclear fluorescence was determined by ImageJ, (*n* = 300 cells per experimental condition), with significant differences determined by Mann–Whitney *U* test. (****p < 0.0001; **p < 0.01). (C) WT and HGPS‐1 fibroblasts were treated with 60 nM Selinex or the vehicle alone for 6,12, 24 h, as well as 2, 3, and 6 days. Cell samples were then immunolabeled for progerin and counterstained with DAPI to decorate nuclei, and representative images from three independent experiments are shown. Bars scale, 10 μM. (D) Lysates from HGPS‐1 cells, grown in MEM (control medium) or KRB medium to induce autophagy; then, cells were treated for 6 h with 50 nM Wortmannin to inhibit autophagy, and further subjected to western blot analysis using antibodies against LC3 and β‐actin (loading control). (E) HGPS‐1 cells grown in KRB medium were treated for 6 h with selinexor (Sel) or the vehicle alone (−), or with both selinexor and Wortmannin (WM), and further by western blotting using specific antibodies for progerin and actin (loading control). *Bottom*. The relative level of progerin was assessed from three independent experiments, using β‐actin as loading control (bottom graph) with significance differences determined by One‐way ANOVA (**p < 0.01). (F) Total RNA isolated from HGPS‐1 cell cultures treated for 6 days with selinexor or the vehicle alone, were subjected to RT‐qPCR analysis to measure progerin mRNA expression. Data were analyzed by unpaired *t*‐test (**p < 0.01).

The clearance of progerin can be induced by drug‐driven activation of autophagy (Cao et al. 2011; Graziotto et al. 2012; Harhouri et al. 2017), thus, owing to the recognized role of selinexor as an autophagy inducer (Silvestrini et al. 2018), we hypothesized whether this compound influences on progerin turnover. A clear depletion of progerin levels was observed in HGPS‐1 cells under treatment with selinexor for 3 days, as shown by WB (~50% decrease) and CLSM (~60% decrease) assays (Figure 4A,B). No signal from progerin was detected in WT cells, validating the specificity of progerin antibodies. Contrarily, progerin levels were refractory to 6 days of treatment with 0.15 or 50 nM LMB (Figure S5). It is worth to note that selinexor treatment appears to preferentially induce a reduction in progerin levels with a significantly lower impact on lamin A/C in both HGPS‐1 and HGPS‐2 fibroblasts (Figure S6). Next, we determined the time‐course of progerin decay in response to selinexor by CLSM analysis. The fluorescence intensity of progerin began to diminish as early as 6 h of treatment and virtually disappeared after 3 days of treatment (Figure 4C). To inquire whether depletion of progerin levels was led by selinexor‐mediated autophagy induction, HGPS cells were treated for 6 h with selinexor or with both selinexor and Wortmannin to inhibit autophagy. Wortmannin is an irreversible inhibitor of phosphatidylinositol 3‐kinase (PI3K‐III), which is required for autophagy initiation via recruitment of other ATG (autophagy‐related genes) proteins at the phagophore (Turkoz Uluer et al. 2021). Thus, autophagy blocking would allow the restoration of progerin basal levels in selinexor treated HGPS fibroblasts. Treatment with Wortmannin prevented the accumulation of LC3‐II, implying that this drug effectively inhibited autophagy (Figure 4D), and consequently, the level of Progerin was recovered (Figure 4E). These results demonstrated a main role for autophagy activation in progerin clearance. Consistently, the level of Progerin mRNA showed non‐significant change upon selinexor treatment, as revealed by qRT‐PCR experiments (Figure 4F).

### 2.4 The Transcriptome Profile and Inferred Cellular Processes Underlying the Anti‐Senescence Effect of Selinexor on HGPS Cells

To determine the gene expression landscape that sustains the geroprotective effect of selinexor on HGPS cells, a high‐throughput RNA sequencing (RNA‐seq) analysis was carried out in WT and HGPS‐1 fibroblasts treated with selinexor or the vehicle for 6 days (see Methods). The RNA‐seq data was obtained from four different experimental conditions (vehicle‐treated WT cells; vehicle‐treated HGPS‐1 cells; selinexor‐treated WT cells and selinexor‐treated HGPS‐1 cells, grown between 5 and 8 passages), each including four biological replicates. We first validated whether the samples showed similar expression levels across replicates (Figure S8A). Next, we examined the samples using principal components analysis (PCA). We found that the experimental conditions form four distinctive groups in the PCA (Figure S8B); the difference between vehicle‐treated and selinexor‐treated explained 73.2% of the variance (PC1) and the difference between WT and HGPS cells explained 22.1% of the variance (PC2). We performed four different differential expression analyses with an FDR < 0.05 (Figure S7). By contrasting vehicle‐treated WT against vehicle‐treated HGPS we found 950 differentially expressed genes (DEGs) in HGPS, 520 genes downregulated, and 430 genes upregulated. In the contrast between vehicle‐treated WT against selinexor‐treated WT, we obtained 447 DEGs, 287 genes downregulated, and 160 genes upregulated by selinexor (Figure S7C). When contrasting vehicle‐treated HGPS against selinexor‐treated HGPS we found 949 DEGs, 590 genes downregulated, and 359 genes upregulated by selinexor. Finally, the contrast between vehicle‐treated WT cells against selinexor‐treated HGPS cells, showed 1280 DEGs, 693 genes downregulated, and 587 genes upregulated by selinexor (Figure S7C). DEGs contained different sets of genes in the comparisons between the WT against either HGPS, selinexor‐treated WT and selinexor‐treated HGPS (Figure S7A), meaning HGPS cells and WT cells are reacting differently to selinexor. The upregulated genes in HGPS, compared to WT, were enriched in biological processes such as extracellular matrix organization, aging, tissue development, etc., whereas genes downregulated in HGPS were enriched in endoderm development (Figure S7B). We found that several genes involved in aging, cell cycle, inflammation, DNA repair, the ribosome, etc. were upregulated in the HGPS phenotype and targeted by selinexor, which downregulates their expression levels (Figure S7E). Particularly, genes linked to aging are downregulated and regained similar levels to the WT condition (Figure S7F).

**FIGURE 5:**
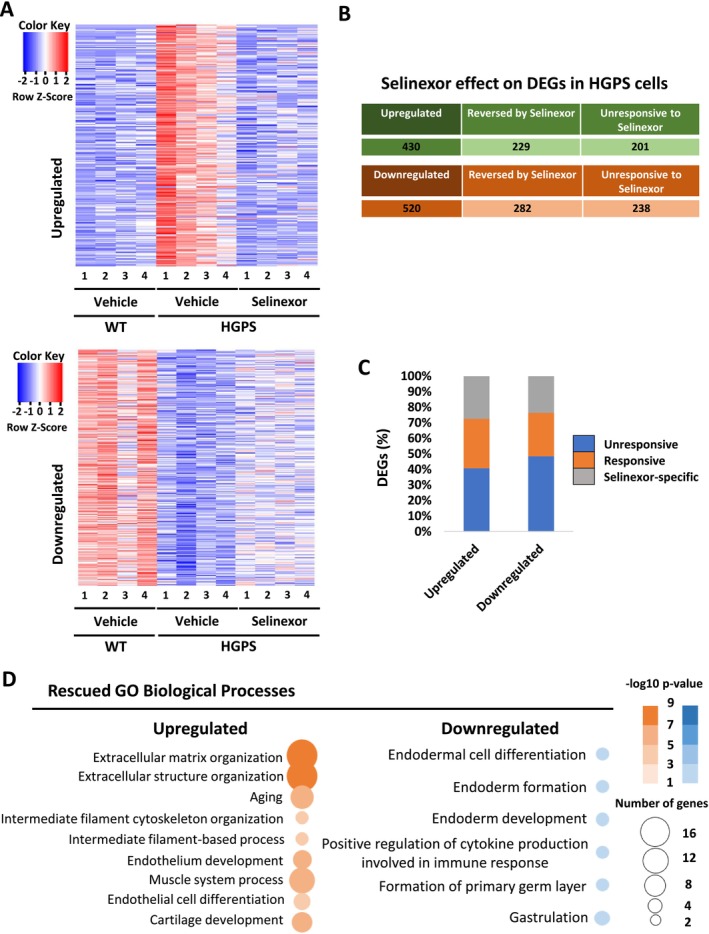
Rescue of DEGs and GO biological processes in selinexor‐treated HGPS fibroblasts. (A) Heat maps illustrating the effect of selinexor on DEGs. (B) Upregulated and downregulated DEGs that were reversed by selinexor. (C) Bar plot showing the percentages of DEGs that were responsive or unresponsive to selinexor, as well as those that were specifically deregulated by selinexor. (D) Rescue of GO biological processes after selinexor treatment.

Importantly, more than half of the genes that showed significant misregulation of their expression levels in the HGPS phenotype regained normal expression levels after selinexor treatment, particularly those genes that were upregulated in HGPS (Figure 5A,B). It is worth to note that a fraction of DEGs were specifically deregulated by selinexor (Figure 5C). Most of the top biological processes that were enriched in HGPS cells were no longer significant after the selinexor treatment, including aging, endoderm development, circulatory system development, and tissue development (Figure 5D). The only biological process that remained enriched after the treatment with selinexor was related to the extracellular matrix organization (Figure 5D), although the FDR values increased from 1.4e‐8 without selinexor to 6e‐3 after selinexor treatment.

### 2.5 Selinexor Treatment Elicits Downregulation of Progerin and Aorta Improvement in *Lmna* ^*G690G/G690G*^ Mice

**FIGURE 6:**
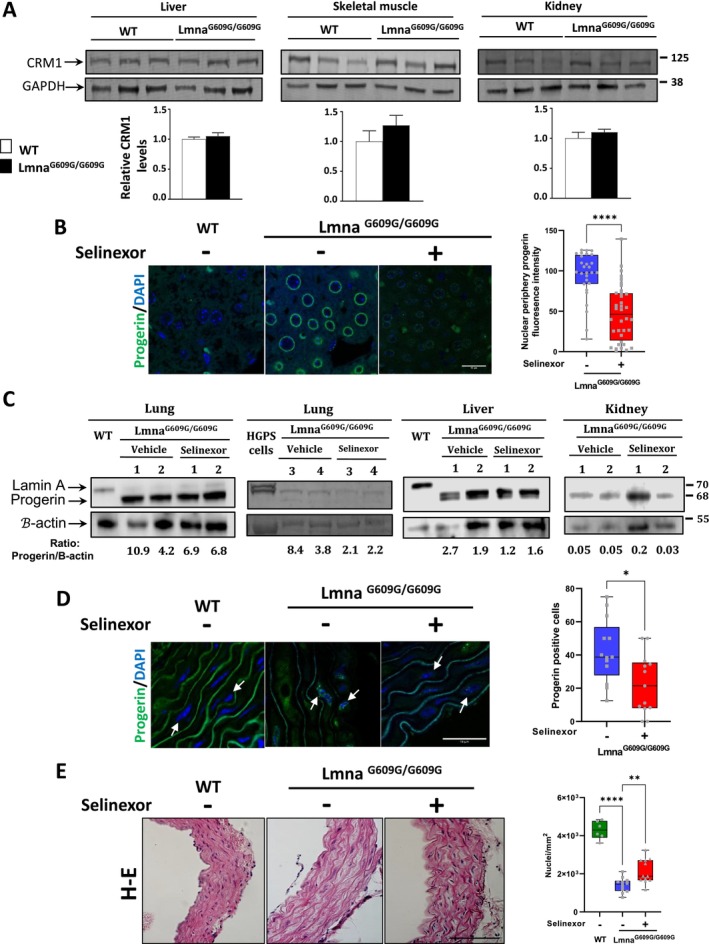
Decreased progerin immunostaining/levels and improved aortic histopathology in the *Lmna* ^*G609G/G609G*^ mice in response to Selinexor treatment. (A) Expression of CRM1 protein in various tissues (liver, skeletal mouse, and kidney) of WT and Lmna^G609G/G609G^ mice. The indicated tissue lysates from WT and progeroid mice were subjected to immunoblotting analysis (see Methods) utilizing specific antibodies for CRM1 and GAPDH (loading control). The mean CRM1 levels in each tissue were obtained from three different WT mice and were set at 1 to compare with those of the three progeroid mice. Data correspond to the mean ± SEM. (B) Liver slices from WT and progeroid mice administrated with selinexor or the vehicle alone for 5 weeks were immunostained for progerin and counterstained with DAPI to visualize nuclei. Typical confocal images with 3D plots are shown (*n* = 3 mice). Bar, 15 μM. Quantification of progerin fluorescence intensity is shown, with significant differences obtained by Mann–Whitney (**** < 0.0001). (C) Tissue lysates (liver, lung, and kidney) from Lmna^G609G/G609G^ mice treated with selinexor or vehicle were subjected to immunoblotting analysis utilizing a specific polyclonal antibody for lamin A and progerin but not lamin C (ab26300), and antibody specific for beta‐actin (loading control). The numbers represent the different mice tested, which were four for the lung analysis and two for the liver and kidney analysis. Lysates from WT mice or HGPS fibroblasts were used as controls. The ratio between progerin and GAPDH was calculated by densitometric analysis of the immunoblots corresponding to each tissue of vehicle‐treated and selinexor‐treated progeroid mice. (D) Aorta sections from WT and progeroid mice administrated with selinexor or the vehicle alone were immunolabeled with progerin antibodies and counterstained with DAPI to decorate nuclei. Representative images from 10 independent aorta sections showing diminished progerin fluorescence in the progeroid mice (*n* = 5 mice per experimental condition) are shown. Scale bar, 15 μm. Right graph shows the quantification of progerin‐positive cells, with each dot representing an independent tissue section (*n* = 5 mice). Significant differences were determined by Mann–Whitney *U* test (*p < 0.05; ** *p* < 0.01;  ****p < 0.0001). (D, E) H‐E staining of aorta slices from WT or *Lmna* ^*G609G/G609G*^ mice treated with selinexor or the vehicle alone. *Right graphs*. Quantification of nuclei per area unit (mm^2^). Data represent the mean ± SEM from 10 independent tissue sections (*n* = 5 mice), with significant differences determined by Mann–Whitney *U* test (*p < 0.01 ***p < 0.0001).

To ascertain whether the elevated level of CRM1 in HGPS fibroblasts is also present in the *Lmna* ^*G609G/G609G*^ mouse (Osorio et al. 2011), tissue lysates from liver, kidney and skeletal muscle from 14‐month‐old WT and progeroid mice were analyzed by immunoblotting assays. The results indicated a tendency for the level of CRM1 to be higher in progeroid mouse tissues, although this difference was not statistically significant (Figure 6A). The beneficial effect of selinexor to counteract progerin was investigated in vivo in the *Lmna* ^*G609G/G609G*^ mouse (Osorio et al. 2011). To determine an effective and low toxic dose of selinexor, WT and *Lmna* ^*G609G/G609G*^ mice were gavaged twice a week with 10 or 2.5 mg/kg selinexor, or the vehicle alone for 5 weeks (6‐ to 11‐week‐old). The doses of selinexor were chosen based on previous experimentation with mice (Abboud et al. 2020; Handley et al. 2021; Muz et al. 2017). Accelerated loss of body‐weight with abrupt drop between 45 and 70 days and shorter healthspan were observed in *Lmna* ^*G609G/G609G*^ mice treated with 10 mg/kg selinexor (Figure S9). In contrast, HGPS mice administered with the lower dose (2.5 mg/kg) lost weight slowly and steadily throughout the treatment, in a similar manner to mice treated with the vehicle alone (Figure S9). Furthermore, HGPS mice administered with the higher dose exhibited less mobility and mild paresis of hind legs, as well as earlier loss of fur and kyphosis, compared to mice treated with 2.5 kg/kg selinexor or the vehicle alone (data not shown). Thus, we chose 2.5 kg/kg selinexor dose for further analyses, as it was well tolerated by the progeroid mice. *Lmna* ^*G609G/G609G*^ mice were treated with selinexor as above and euthanized at the endpoint, then liver and aorta tissues were subjected to immunohistological analyses. Interestingly, a significant reduction in the immunostaining of progerin was evoked in the liver after selinexor administration, compared to vehicle‐treated samples (Figure 6B). The impact of selinexor administration on the levels of progerin was also assessed by immunoblotting assays in different progeroid mice tissues (lung, liver, and kidney). A polyclonal antibody that specifically detects lamin A and progerin, but not lamin C (ab26300), was utilized. A decrease in the level of progerin was observed in the selinexor‐treated mice in most of the tissue samples analyzed (Figure 6C). In alignment with the IF results, liver samples from the two progeroid mice displayed a reduction in progerin levels. Owing that cardiovascular dysfunction is a hallmark of HGPS (Cisneros et al. 2023; Gerhard‐Herman et al. 2012; Rahman et al. 2021), we sought to evaluate the ability of selinexor to improve *Lmna* ^*G609G/G609G*^ mouse aortic histopathology. We found a significant decrease of progerin immunostaining in the aorta sections of selinexor‐treated progeroid mice, compared with vehicle‐treated animals (Figure 6D). Remarkable, the loss of vascular smooth muscle cells (VSMCs), a main feature of HGPS vasculature (Hamczyk and Nevado 2023; Hamczyk et al. 2018; Kang et al. 2023), was partiality prevented/reversed in selinexor‐fed HGPS mice, as shown by the increased number of nuclei per area unit in the hematoxylin–eosin (H‐E)‐stained aorta slices (Figure 6E). These results imply that progerin decline is functionally linked to the alleviation of aortic histopathology.

### 2.6 For Materials and Methods See Supporting Information

#### 2.6.1 Statistical Analysis

Statistical analyses were performed using GraphPad Prism 8 software (San Diego) by the two‐tailed unpaired Student's *t*‐test. Data represent the mean ± SEM from three independent experiments, and *p* < 0.05 are indicative of statistical significance. Where indicated, statistical analyses were performed using exact nonparametric Mann–Whitney *U* test or unpaired *t*‐test, and data were represented by the mean ± SEM or the mean ± SD from a series of three independent experiments, *p* < 0.05 were considered as significant.

## 3 Discussion

As aging progresses, the functioning of proteostasis‐associated mechanisms decays, including protein folding, nuclear protein trafficking and the two main pathways governing protein turnover, namely 26S proteasome and autophagy (López‐Otín et al. 2023). Specifically, dysfunction of nuclear protein transport due to impaired cycles of import and export results in unbalanced partitioning of proteins between the nucleus and the cytoplasm, which in turn disturbs a plethora of cellular processes, including DNA replication and repair, gene expression, cell cycle, ribosomal biogenesis, as well as pathways associated with aging, such as cellular senescence, telomere stability and mitochondrial function (Kumar and Lapierre 2021; Mor, White, and Fontoura 2014; Park et al. 2021). Indeed, defective nucleocytoplasmic trafficking of proteins is the molecular basis of different aging‐linked diseases including cancer (Stelma et al. 2016), neurodegeneration (Girdhar and Guo 2022), and premature aging (Kumar and Lapierre 2021; Martins et al. 2020; Park et al. 2021). In previous work, we established that primary fibroblast from patients with HGPS exhibited abnormal enhancement of CRM1‐mediated nuclear protein export, and that this alteration is intimately related to their senescent phenotype (García‐Aguirre et al. 2019). We demonstrated that pharmacological blocking of CRM1 using LMB mitigated an array of senescent marks in HGPS fibroblasts, and consistent with a key role for the CRM1‐mediated nuclear export in HGPS, overexpression of CRM1 lead rapidly normal fibroblasts to senescence (García‐Aguirre et al. 2019). In this scenery, it is relevant and timely to determine whether pharmacological modulation of CRM1 could be a viable therapeutic strategy against HGPS. To approach this, it is necessary to select a specific inhibitor of CRM1 with superior pharmacological properties than LMB, which is highly toxic in vivo (Newlands, Rustin, and Brampton 1996), and to evaluate treatment efficiency not only in cultured HGPS fibroblasts but also in an HGPS organism model.

In this study, we demonstrate that treatment of HGPS fibroblasts with selinexor, first in class selective inhibitor of CRM1 developed by Karyopharm Therapeutics (Gravina et al. 2015; Ishizawa et al. 2015; Landes et al. 2023) alleviates a number of senescence markers in HGPS fibroblasts, including aberrant nuclear morphology, senescent cellular morphology, nucleolar expansion, loss of heterochromatin, aberrant mitochondrial morphology and increased ROS production, but not lamin B1 downregulation neither increased SA‐β‐gal activity. Furthermore, selinexor elicited downregulation of p16^INK4A^ and p21^CIP/WAF1^, and decreased the percentage of cells at the G0/G1 phase in HGPS fibroblasts, confirming its anti‐senescent activity at a biochemical level. Although the level of lamin B1 was not recovered by the selinexor treatment, there was a restoration of the heterochromatin mark H3K9m3. This may be attributed to the positive regulation exerted by selinexor on other critical components that maintain this repressive mark, including SUV39H1 (the major H3K9me3 histone methyltransferase) and HP1α (a protein that recognizes and binds to H3K9me3 to form a stable heterochromatin complex) (Padeken, Methot, and Gasser 2022); however, further experiments are required to confirm this hypothesis. The critical role of heterochromatin loss on the senescent phenotype of HGPS cells has been previously determined (Columbaro et al. 2005). These authors demonstrated that a combined treatment of HGPS cells with mevinolin (a farnesyl‐transferase inhibitor) and Trichostatin A (TSA, an inhibitor of histone deacetylase activity) resulted in the restoration of both heterochromatin organization and intranuclear distribution, as well a reduction of progerin levels. Furthermore, heterochromatin loss has been linked to mitochondrial dysfunction and DNA damage, as reviewed in (Lee et al. 2020). Thus, it can be reasonably inferred that the recovery of heterochromatin in response to selinexor treatment may be a significant event that effectively prevents the subsequent cascade of alterations that are characteristic of a senescent cell. On the other hand, owing that transcription factor EB (TFEB) is and client of CRM1 (Napolitano and Ballabio 2016) and a master regulator of lysosomal function (Napolitano et al. 2018), it can be logically deduced that the augmented SA‐β‐gal activity observed in selinexor‐treated HGPS cultures, was attributed to the enhanced lysosomal activity promoted by the nuclear accumulation of TFEB, rather than indicative of the presence of senescent cells. Selinexor showed much lower toxicity than LMB, as determined by their respective IC50 values. Selinexor acts by binding selectively to the Cys 528 residue of CRM1, thereby inhibiting CRM1 binding to the NES domains in the cargo‐binding groove (Ishizawa et al. 2015). Unexpectedly, treatment with selinexor but not with LMB could induce progerin clearance in human HGPS fibroblasts and importantly in the *Lmna* ^*G609G/G609G*^ mouse (see below).

Nuclear export dysfunction due to increased expression/activity of CRM1 is a hallmark shared by HGPS and different types of cancer (García‐Aguirre et al. 2019; Mahipal and Malafa 2016); however, pharmacological inhibition of CRM1 mediated by selinexor has different physiological consequences in each cell system. In cancer cells, treatment with selinexor provokes the nuclear accumulation and enhanced activity of many tumor suppressor factors, including p21^CIP/WAF1^, p27^KIP1^, p53, p73, BRCA 1/2, RB, and FOXO proteins (Mahipal and Malafa 2016). In addition, selinexor leads to nuclear sequestering of eIF4E‐oncogene mRNA complexes, resulting in decreased translation of MYC, BCL2, and BCL6 oncogenes (Cisneros and García‐Aguirre 2020). Collectively these events culminate in the apoptosis of cancer cells, while normal cells remain unaffected (Gravina et al. 2015; Mahipal and Malafa 2016). Based on the strong and selective antitumoral activity of selinexor, it is currently undergoing study in about 60 clinical trials, including sarcomas, gliomas, leukemias, gastric cancer, melanoma, and colorectal cancer (Gravina et al. 2015). On the contrary, selinexor treatment rescues the pathological phenotype of HGPS cells with no apparent induction of apoptosis, likely by trapping in the nucleus NES‐containing proteins whose function can counteract cellular senescence (García‐Aguirre et al. 2019). Among the protein clients of CRM1 that might attenuate senescence marks are Sirtuin 2, involved in heterochromatin organization; B23, implicated in nucleoli structure and function; dystrophin Dp71 and β‐dystroglycan, involved in nuclear architecture; telomerase, essential for telomere homeostasis; and p53, critical for cell cycle progression and replicative senescence (Cisneros and García‐Aguirre 2020). A recent article demonstrated that EP300, a lysine acetyltransferase critically involved in the mTORC1/autophagy axis regulation, is mislocalized to the cytoplasm in HGPS fibroblasts due to CRM1 hyperactivity. Interestingly, normalization of EP300 nuclear localization resulted in HGPS alleviation (Son et al. 2024). Further proteomic analysis to determine whether these and/or other CRM1 protein clients associated with senescence undergo nucleocytoplasmic redistribution in response to selinexor is warranted.

Noteworthy, the effect of selinexor on cellular senescence was also reflected at the transcriptional level, as it diminished the expression of numerous genes that were previously found to be upregulated in HGPS fibroblasts, enriching aging‐associated cellular processes, including cell cycle, inflammation, ribosome function, autophagy, and DNA repair. In alignment with our results, the beneficial effect of silencing CRM1 expression on longevity was previously shown in nematodes (Buchwalter and Hetzer 2017; Silvestrini et al. 2018). In this system, the genetic or pharmacological blocking of CRM1 leads to the relocation of nuclear proteins and consequently to autophagy induction, which in turn reduces protein synthesis and alleviates nucleolar expansion, via downregulation of RPL‐11 (ribosomal protein) and fibrillarin (nucleolar protein) respectively. These authors propose that attenuation of CRM1 activity improved proteostasis and extend lifespan through a mechanistic link between autophagy‐mediated protein turnover, ribosome dynamics, and protein translation (Silvestrini et al. 2018).

Apart from the benefit of relocating key proteins to the nucleus, selinexor promotes progerin clearance, which in turn must ameliorate downstream toxic effects exerted by the mutant protein. In this regard, some compounds with the ability to induce progeria degradation through autophagy activation have been previously identified, including rapamycin (inhibitor of mTOR pathway) (Graziotto et al. 2012), MG132 (proteasome inhibitor) (Harhouri et al. 2017), sulforaphane (antioxidant compound) (Gabriel et al. 2015) and neuropeptide Y (Aveleira et al. 2020). In a similar manner, selinexor appears to decrease progerin content by activating autophagy with no apparent effect on its corresponding mRNA. Thus, the antisenescence action in HGPS occurs through at least two distinct mechanisms: (a) restoration of the nucleocytoplasmic balance of proteins by attenuating CRM1 activity and (b) cutting down the detrimental action of progerin by promoting progerin depletion. In regard to the second assertation, given that lamin A plays a role in nuclear protein trafficking through its interaction with nuclear pore proteins such as Nup153 and Nup155 (Han et al. 2019; Scott and Parekh 2022), it is likely that the aberrant binding between progerin and lamin A (Lee et al. 2016) impairs this cellular process. Therefore, the decrease in progerin caused by selineor treatment would facilitate the release of lamin A, thereby enabling normalization of nuclear protein transport. While progerin clearance mediated by selinexor may be sufficient to improve the physiology of HGPS cells, we believe that the nuclear accumulation of key proteins due to CRM1 inhibition, contributes to the therapeutic effect of Selinexor for the following reasons. (a) enforced overexpression of CRM1 causes cellular senescence in WT fibroblasts lacking progerin (García‐Aguirre et al. 2019), indicating that proper nucleo‐cytoplasmic distribution of proteins is necessary to prevent this process, and (b) treatment with selinexor mitigates the senescent markers in primary fibroblasts from aged individuals, which lack progerin (unpublished data).

We assessed the therapy of inhibiting CRM1 with selinexor in the *Lmna* ^*G609G/G609G*^ mouse (Osorio et al. 2011), Our findings indicate that the level of CRM1 tends to be elevated in various tissues of the progeroid mouse; nevertheless, additional research is necessary to determine whether there is a mechanistic correlation between CRM1 levels and both aging and progerin abundance. Regarding the in vivo efficacy of selinexor, our results showing a decrease of progerin immunostaining in the liver and aorta, and a decrease of progerin levels by immunoblotting in selinexor‐treated progeroid mice in most tissue samples, as well as an improvement of the aortic histopathology in the selinexor‐treated progeroid mice are promising. However, differences between immunoblotting experiments, which might be due to tissue‐specific or individual‐specific effects were found. A comprehensive analysis with simultaneous monitoring of the progerin level and autophagy status in disease‐sensitive organ (Hasper et al. 2024) is required to ensure that selinexor triggers autophagy and consequently facilitates progerin degradation. Furthermore, it is essential to determine whether selinexor therapy can extend the lifespan of the progeria mouse model. So far, the therapeutic use of selinexor has specifically been for cancer in xenograft mouse models (Landes et al. 2023) and even in clinical trials (Abdul Razak et al. 2016; Lassman et al. 2022). Therefore, the present study showing the gero‐protective effect of selinexor in human cells and a murine model will serve as a platform to expand the study of selinexor in the therapeutic of aging and its chronic degenerative associated disorders.

In summary, we showed that pharmacological inhibition of CRM1 using selinexor is a feasible strategy to treat HGPS and demonstrated that the robust anti‐senescent activity of selinexor is exerted by recovering proper nucleocytoplasmic partition of proteins and inducing progerin clearance in HGPS fibroblasts. The proven ability of selinexor to decrease progerin in vivo in the *Lmna* ^*G609G/G609G*^ mice as well as the current exhaustive study of selinexor in cancer trials, would pave the way for future therapeutic intervention in aging.

## Author Contributions

**Bulmaro Cisneros**, **Susana Castro‐Obregón**, **Diego Cortez**, **Adriana Soto‐Ponce**, and **Jonathan J. Magaña:** conceptualization. **Adriana Soto‐Ponce**, **Ian García‐Aguirre**, **Marlon De Ita**, **Juan Unzueta**, **Isabel Arrieta‐Cruz**, **Tania Zavaleta** and **Diego Cortez:** data curation. **Adriana Soto‐Ponce**, **Ian García‐Aguirre**, **Marlon De Ita**, **Juan Unzueta**, **Isabel Arrieta‐Cruz**, **Tania Zavaleta**, and **Diego Cortez:** formal analysis. **Bulmaro Cisneros**, **Susana Castro‐Obregón**, **Diego Cortez**, **Yosef Landesman**, **Jonathan J. Magaña**, and **Isabel Arrieta‐Cruz:** funding acquisition. **Bulmaro Cisneros**, **Adriana Soto‐Ponce**, **Ian García‐Aguirre**, **Marlon De Ita**, **Juan Unzueta**, **Isabel Arrieta‐Cruz**, **Tania Zavaleta**, **Isabel Arrieta‐Cruz**, **Angelica Soberano‐Nieto**, **Porfirio Nava**, **Aurora Candelario‐Martínez**, and **Diego Cortez:** investigation. **Bulmaro Cisneros**, **Isabel Arrieta‐Cruz**, **Jonathan J. Magaña:** project azdministration. **Bulmaro Cisneros**, **Susana Castro‐Obregón**. **Diego Cortez**, **Yosef Landesman**, **Jonathan J. Magaña**, and **Isabel Arrieta‐Cruz:** resources. **Bulmaro Cisneros**, **Isabel Arrieta‐Cruz**, **Jonathan J. Magaña:** supervision. **Bulmaro Cisneros**, **Isabel Arrieta‐Cruz**, **Jonathan J. Magaña:** visualization. **Bulmaro Cisneros**, **Susana Castro‐Obregón**, **Susana Gonzalo**, **Diego Cortez**, **Ian García‐Aguirre**, **Marlon De Ita**, and **Jonathan J. Magaña:** writing – original draft preparation. **Bulmaro Cisneros**, **Susana Castro‐Obregón**, **Susana Gonzalo**, **Diego Cortez**, **Ian García‐Aguirre**, **Marlon De Ita**, and **Jonathan J. Magaña:** writing – review and editing.

## Ethics Statement

The experimental procedures were conducted in concordance with the Institutional Animal Use and Care guideless (Mexican official norm, NOM‐062‐ZOO‐1999), and the corresponding approved protocol (No. 0314‐20), Laboratory Animals Unit (UPEAL‐CINVESTAV, CDMX, Mexico).

## Conflicts of Interest

The authors declare no conflicts of interest.

## Supporting information


Data S1.

## Data Availability

The data that support the findings of this study are available from the corresponding author upon reasonable request.
