# Supplementary material for: Targeting CRM1 for Progeria Syndrome Therapy
Source: Aging Cell. 2025 Jan 27;24(5):e14495. doi: 10.1111/acel.14495 (PMC12073922; doi:10.1111/acel.14495)
Supplement: Supplementary file 1 — Data S1. [file ACEL-24-e14495-s001.pdf]

Supplementary information

Targeting CRM1 for progeria syndrome therapy

Adriana Soto-Ponce[1¶], Marlon De Ita[1,2¶], Susana Castro-Obregón[3], Diego Cortez[4], Josef Landesman[5], Jonathan J. Magaña[6,7], Susana Gonzalo[8], Tania Zavaleta[1], Angelica Soberano-Nieto[1] Juan Unzueta[9], Isabel Arrieta-Cruz[10], Porfirio Nava[11], Aurora Candelario-Martínez[11], Ian García-Aguirre[7,*], Bulmaro Cisneros[1,*]

[1]Departamento de Genética y Biología Molecular, Centro de Investigación y de Estudios Avanzados, Ciudad de México 07360, México.
[2]Unidad de Investigación Médica en Genética Humana, Hospital de Pediatría, Centro Médico Nacional Siglo XXI, IMSSS, Ciudad de México 06720, México.
[3]Instituto de Fisiología Celular, UNAM, Ciudad Universitaria, CP04510 Ciudad de México, México
[4]Centro de Ciencias Genómicas, UNAM, CP62210 Cuernavaca, México.
[5]Karyopharm Therapeutics, Newton, MA 02459, USA.
[6]Laboratorio de Medicina Genómica, Departamento de Genética (CENIAQ), Instituto Nacional de Rehabilitación-Luis Guillermo Ibarra Ibarra (INR-LGII), Ciudad de México 14389, Mexico.
[7]Departamento de Bioingeniería, Escuela de Ingeniería y Ciencias, Tecnologico de Monterrey, Ciudad de México 14380, Mexico.
[8]Edward A. Doisy Department of Biochemistry and Molecular Biology, Saint Louis University School of Medicine, St. Louis, MO, USA.
[9]Universidad Autónoma Metropolitana, Unidad Iztapalapa, División de Ciencias Biológicas y de la Salud.
[10]Departamento de Investigación Básica, División de Investigación, Instituto Nacional de Geriatría, Secretaría de Salud, Ciudad de México 10200, México.
[11]Departamento de Fisiología, Biofísica y Neurociencias, Centro de Investigación y de Estudios Avanzados, Ciudad de México 07360, México.

[*]Corresponding information:

Bulmaro Cisneros Vega: bcisnero@cinvestav.mx

Ian Alain García Aguirre: ian.garcia@tec.mx

¶These authors contributed equally to this work.

Keywords: Aging, Progeria, Senescence

This file includes:

- **Supplemental Figure 1.** Treatment of HGPS cells with LMB induced the nuclear accumulation of cyclin B1 but failed to downregulate CRM1 protein levels.
- **Supplemental Figure 2.** Analysis of apoptosis and cell cycle in selinexor-treated HGPS fibroblasts.
- **Supplemental Figure 3.** Morphometric analysis of nuclei upon treatment of HGPS fibroblasts with selinexor or LMB.
- **Supplementary Figure 4.** Treatment of HGPS cells with a $>IC_{50}$ dose of LMB but not with a $<IC_{50}$ dose mitigated the senescent features of HGPS cells.
- **Supplemental Figure 5.** Progerin levels were unaffected by LMB treatment.
- **Supplemental Figure 6.** Treatment with selinexor resulted in a preferential reduction in progerin levels in HGPS-1/2 cells.
- **Supplemental Figure 7.** Differentially expressed genes (DEGs) of HGPS fibroblasts and functional enrichment analysis in response to selinexor.
- **Supplemental Figure 8.** Raw data from RNA-seq analysis.
- **Supplemental Figure 9.** Body weight changes in the *Lmna*$^{G609G/G609G}$ mice over the course of selinexor treatment.
- **Materials and methods.**

Supplementary material

Supplementary 1

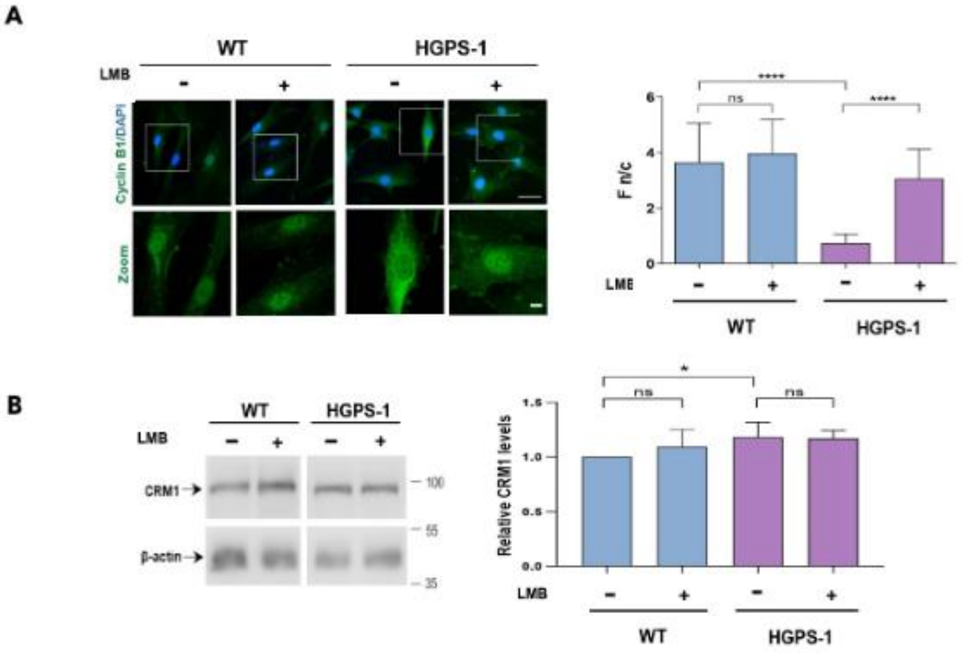

Supplemental Figure 1. Treatment of HGPS cells with LMB induced the nuclear accumulation of cyclin B1 but failed to downregulate CRM1 protein levels. A) WT and HGPS-1 cells seeded on coverslips were incubated with 0.15 nM LMB or the vehicle alone, prior to be immunolabeled for cyclin B1 and counterstained with DAPI to visualize nuclei. Cell preparations were analyzed by CLSM and typical images are shown (bar, 10 µM). *Right*. Significant differences of the cyclin B1 Fn/c ratio of were determined by Mann–Whitney U, with 300 cells analyzed per experimental condition (**** $p < 0.0001$; ns, no significance). (B) Lysates from WT and HGPS-1 cell treated as per A were subjected to western blotting analysis using antibodies against CRM1 and actin (loading control). *Right*. The protein level of CRM1 was estimated from three separate assays, and significant differences were obtained by unpaired t test (* $p < 0.05$; ns, no significance).

Supplementary 2

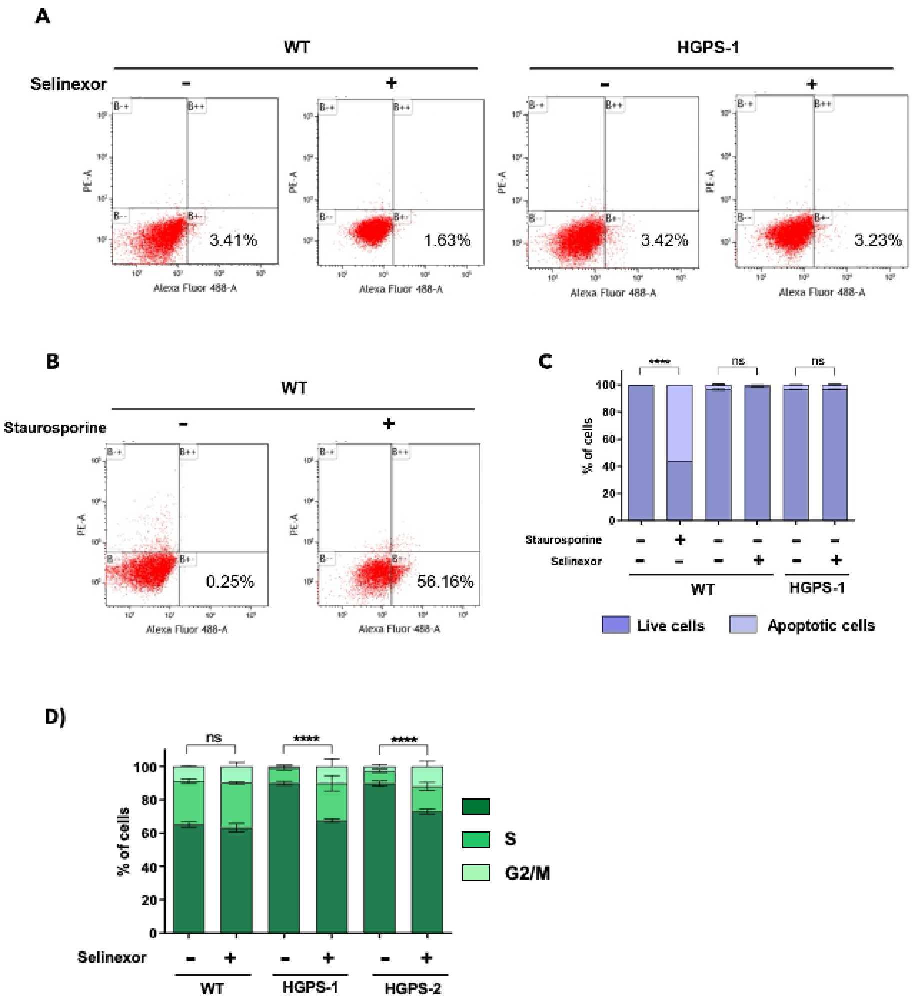

Supplemental Figure 2. Analysis of apoptosis and cell cycle in selinexor-treated HGPS fibroblasts. (A) WT and HGPS-1 cells were treated with selinexor or the vehicle alone for six days (A) or with 1 µM staurosporine for 12 h (B), prior to being stained with Annexin V and propidium iodide (PI) and further analyzed by flow cytometry. The percentage of live and apoptotic cells (positive for both Annexin V and PI can be seen in the bottom left quadrant and the bottom right quadrant respectively. (C) The percentage of live and apoptotic cells were calculated from three separate experiments with significant differences determined by Mann–Whitney U test (****$p < 0.0001$; ns, no significance. (D) Asynchronic cultures of WT and HGPS-1/HGPS-2 cells were treated for 6 days with 60 nM selinexor or the vehicle alone, and their cell cycle profile was further analyzed by flow cytometry, to calculate the percentage of cell populations at each cell cycle phase. Significant differences were determined from three independent experiments using unpaired t test (**** $p < 0.0001$; ns, no significance).

Supplementary 3

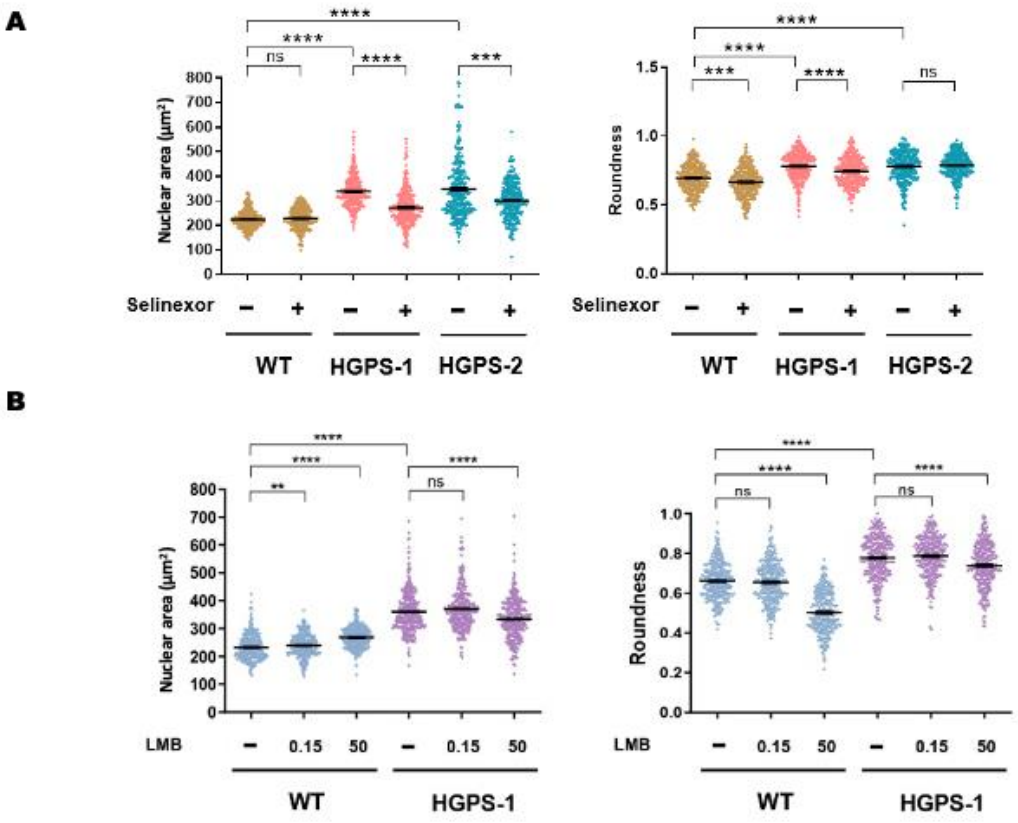

Supplemental Figure 3. Morphometric analysis of nuclei upon treatment of HGPS fibroblasts with selinexor or LMB. WT and HGPS-1/2 cells were treated for 6 days with 60 nM selinexor or the vehicle alone (A), or with 0.15 nM (<IC_{50} dose) or 50 nM (>IC_{50} dose) LMB, or the vehicle alone (B), prior to be immunolabeled for lamin A/C and counterstained with DAPI to measure nuclear area and roundness using Image J software. Significant differences were determined by Mann–Whitney U test (n=300 nuclei per experimental condition; ****$p< 0.0001$; ***$p< 0.001$; ns, no significance).

Supplementary 4

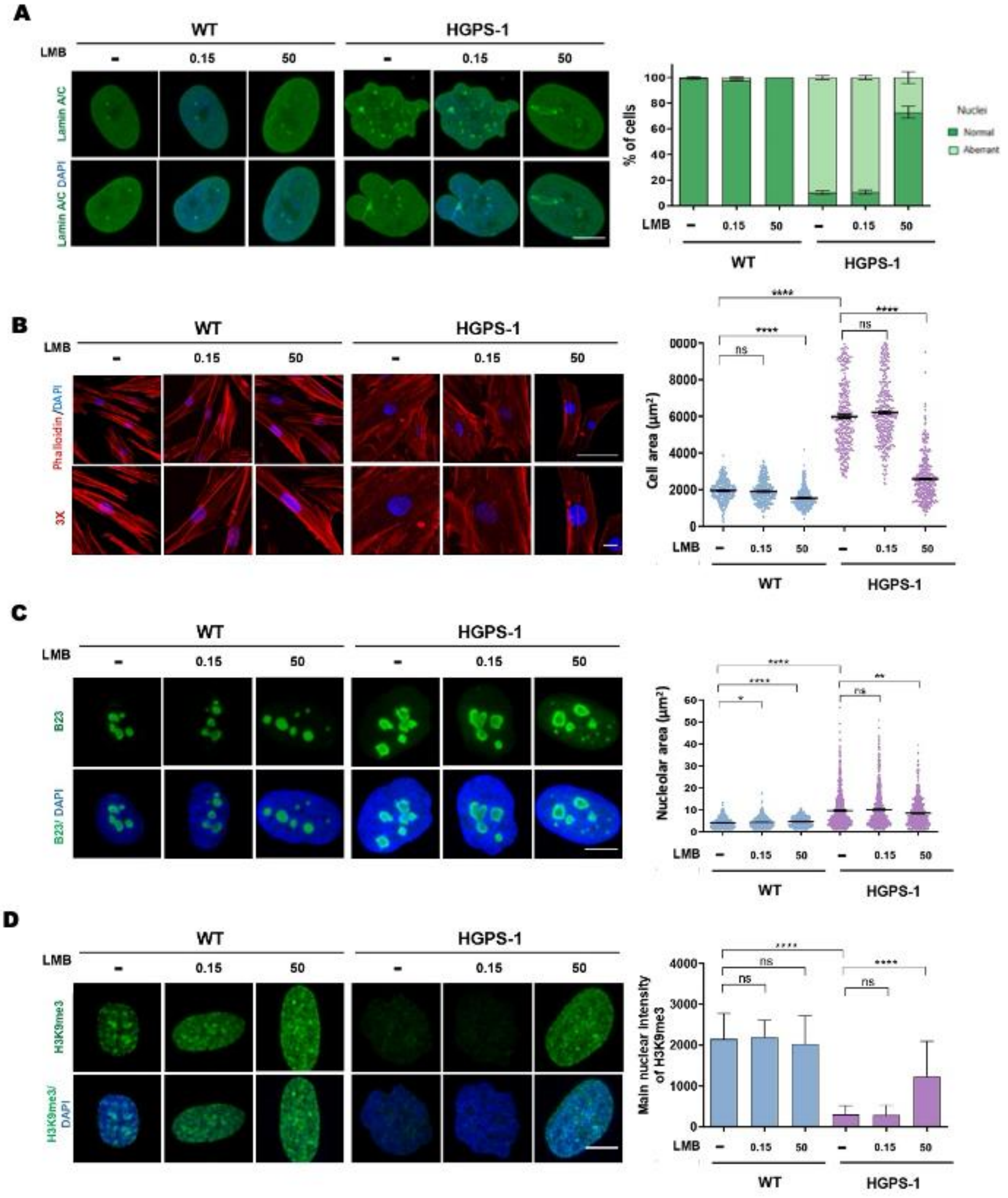

Supplementary Figure 4. Treatment of HGPS cells with a $>IC_{50}$ dose of LMB but not with a $<IC_{50}$ dose mitigated the senescent features of HGPS cells. WT and HGPS-1 fibroblasts cultured on coverslips were incubated for 6 days with 0.15 nM ($<IC_{50}$ dose), or 50 nM ($>IC_{50}$ dose) LMB, or the vehicle alone, prior to be analyzed by CLSM. (A) Cells were immunolabeled for lamin A/C and counterstained with DAPI to analyze nuclear shape. The graph on the right shows the percentage of normal (oval-shaped nuclei) and irregularly shaped nuclei. (B) Cells were labeled with phalloidin to decorate the actin-based cytoskeleton and evaluate cell morphology, (C) Cells were immunolabeled with B23 antibodies to assess the nucleolar area. (D) To visualize heterochromatin, cells were immunostained with antibodies against histone H3K9m3. (A-D) Representative images from three separate experiments are shown: bar scale, 10 μM. Data were collected from three separate experiments (n =300 cells per condition), with significant differences determined by Mann–Whitney U test (****$p < 0.0001$; **$p < 0.01$, ns, no significance).

Supplementary 5

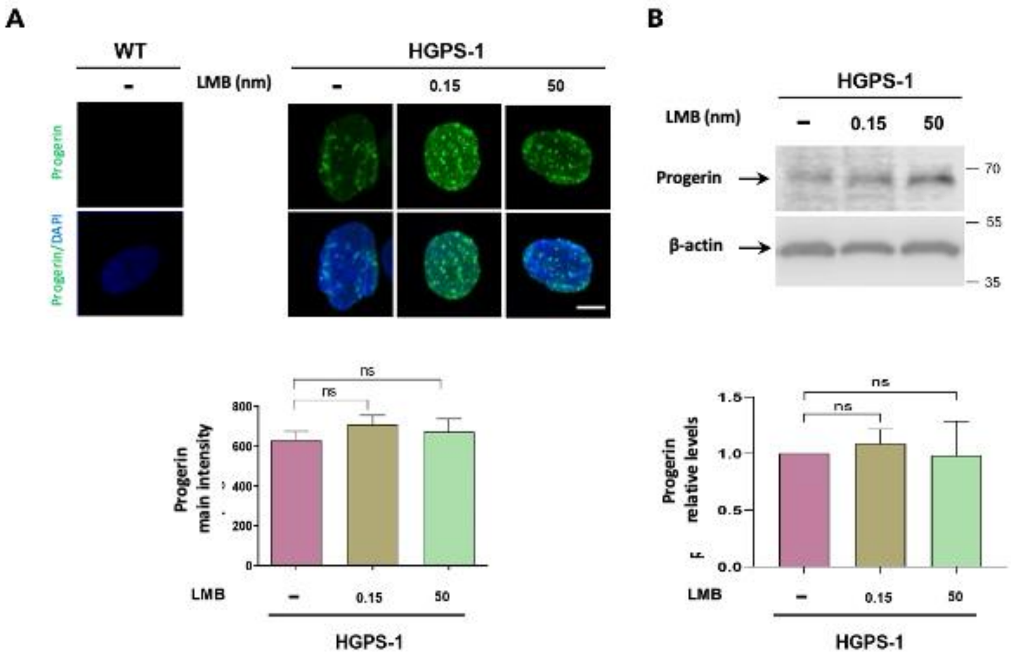

Supplemental Figure 5. Progerin levels were unaffected by LMB treatment. (A) WT and HGPS-1 fibroblasts grown on coverslips were treated for 6 days with 0.15 nM, 50nM LMB or the vehicle alone (-), prior to be immunolabeled for progerin and counter stained with DAPI to decorate nuclei. Representative images from three separate assays are shown. Bar scale,10µM. *Bottom*. The main nuclear fluorescence was determined by Image J (n=300 cells per experimental condition), with no significant differences (ns) between the different experimental conditions determined by Mann–Whitney U test. (B) Lysates from WT and HGPS-1 cells treated for 6 days with 0.15 nM or 50 nM LMB, or the vehicle alone, were analyzed by western blotting using antibody against progerin and actin (loading control). *Bottom*. Relative progerin levels were calculated from three independent experiments, with no significant differences (ns) between the different experimental conditions determined by unpaired t test.

Supplementary 6

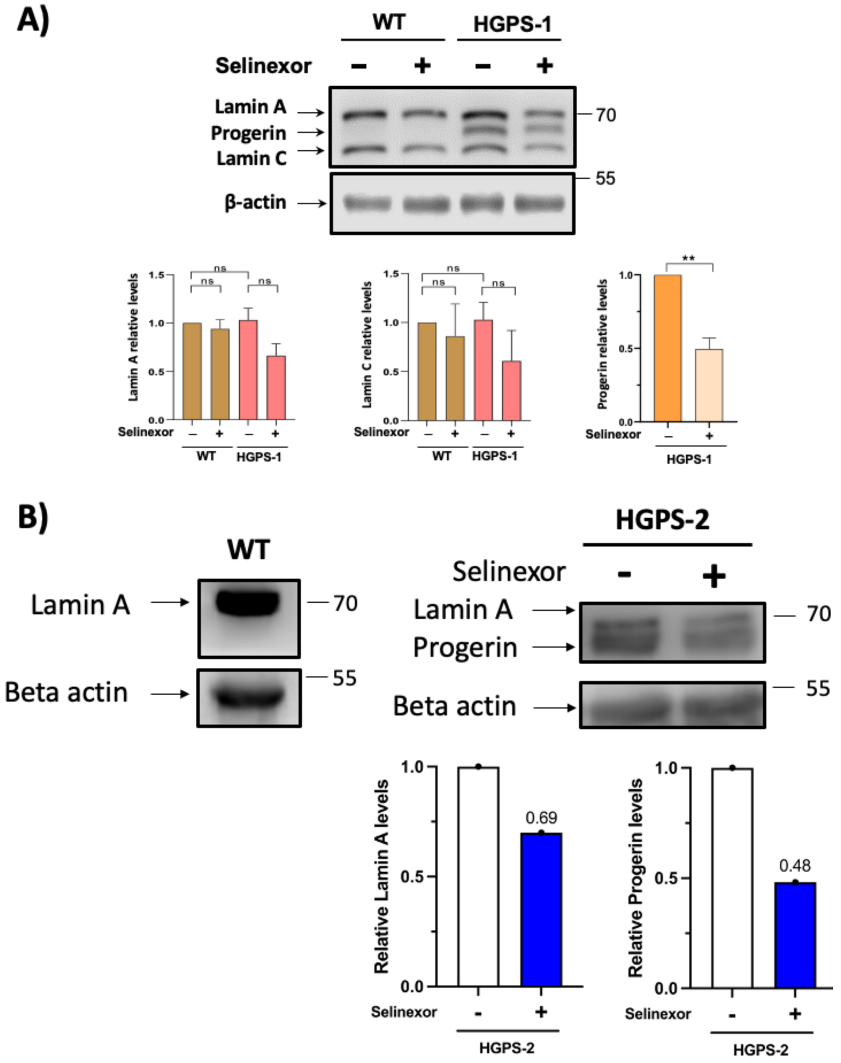

Supplemental Figure 6. Treatment with selinexor resulted in a preferential reduction in progerin levels in HGPS-1/2 cells. (A) Lysates from WT and HGPS-1 fibroblasts cultures treated for 6 days with 60 nM selinexor or the vehicle alone were analyzed by immunoblotting using a pan-antibody against lamin A/C and progerin and anti-actin antibody (loading control). The relative levels of lamin A. lamin C and progerin were assed from three separate experiments, with significant differences determined by unpaired t student (** *p*< 0.0; ns, no significance). (B) Lysates from WT fibroblasts (*right*) and from HGPS-2 fibroblasts treated for 6 days with 60 nM selinexor or the vehicle alone were analyzed by immunoblotting using a polyclonal antibody specific for lamin A and progerin but not lamin C (ab26300), and anti-actin antibody (loading control). A typical immunoblotting image form two independent experiments is shown. The graphs below illustrate the quantification of the relative levels of lamin A and progerin.

Supplementary 7

A

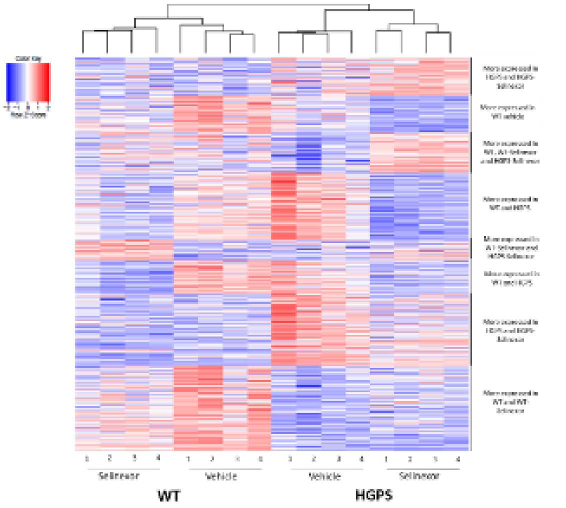

B

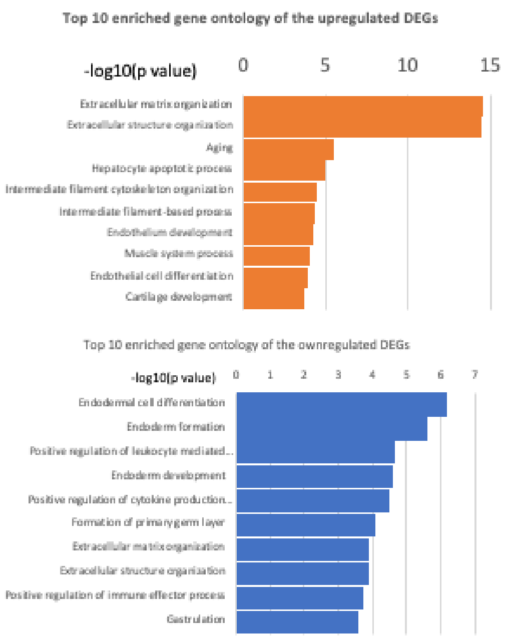

C

WT vs HPGS	DEGs
Downregulated	520
Upregulated	430

WT vs WT-Selinexor	DEGs
Downregulated	287
Upregulated	160

HGPS vs HGPs-Selinexor	DEGs
Downregulated	590
Upregulated	359

WT vs HPGS-Selinexor	DEGs
Downregulated	693
Upregulated	587

D

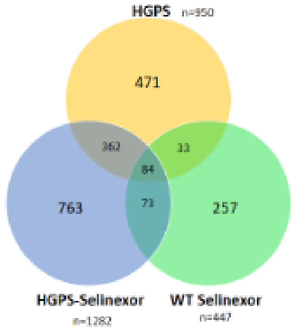

E

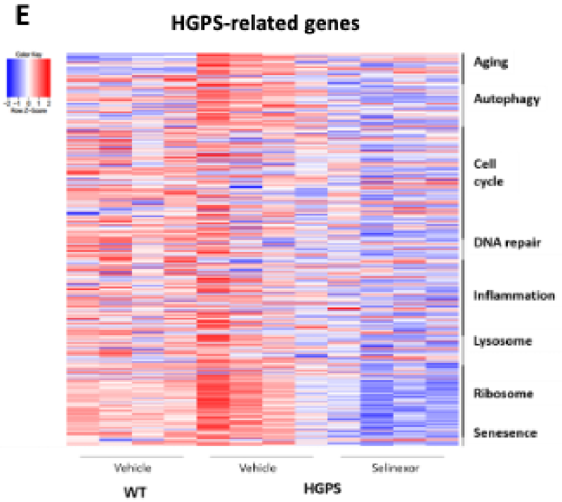

F

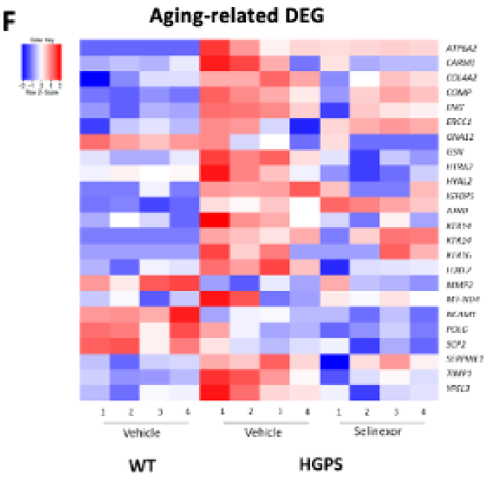

Supplemental Figure 7. **Differentially expressed genes (DEGs) of HGPS fibroblasts and functional enrichment analysis in response to selinexor.** (A) Heat map illustrating the transcriptome profile of WT and HGPS cells treated with selinexor or the vehicle alone, and hierarchical clustering of DEGs. (B) Gene ontology (GO) enrichment analysis to identify the top ten enriched biological processes for upregulated or downregulated selinexor-responsive genes. (C) Downregulated and upregulated DEGs were identified by contrasting the indicated pairs of experimental groups. (D) Venn diagram illustrating a comparison of DEGs: WT versus HGPS, HGPS-selinexor, and/or WT-selinexor. (E) Heat map illustrating DEGs associated with biological processes relevant to HGPS. (F) Heat map showing selinexor-responsive genes linked to aging.

Supplementary 8

A

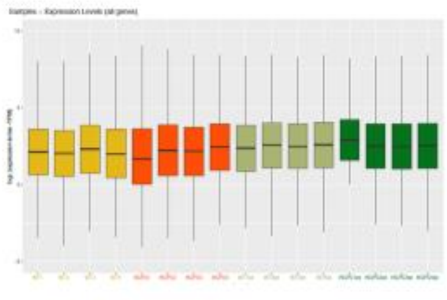

B

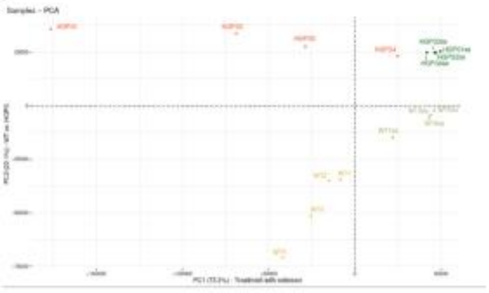

C

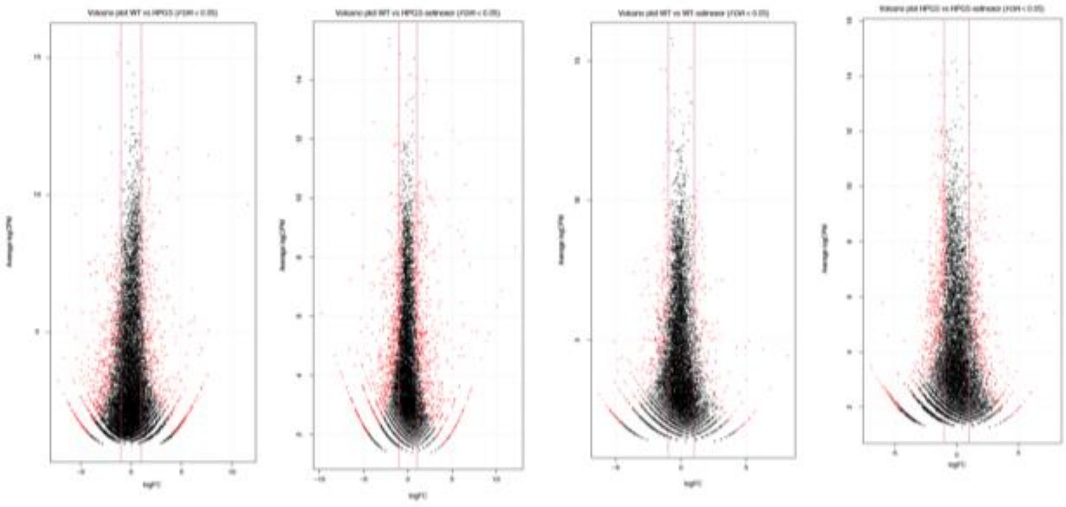

Supplemental Figure 8. Raw data from RNA-seq analysis. A) Validation of gene expression data across experimental replicates. B) Principal components analysis (PCA). The plot spans the samples on a 2-dimensional space illustrating the clustering of biological replicates and the distances between WT and HGPS-1 cells and between vehicle-treated and selinexor-treated cells. C) Vocano plots visualizing differentially expressed genes (DEGs) identified in the four selected contrasts (WT vs HGPS; WT vs HGPS-selinexor; WT vs WT-selinexor and HGPS vs HGPS-selinexor. Red dotes indicate down-regulated (left) and up-regulated (right) genes, while black dots denote genes with no significant changes in gene expression.

Supplementary 9

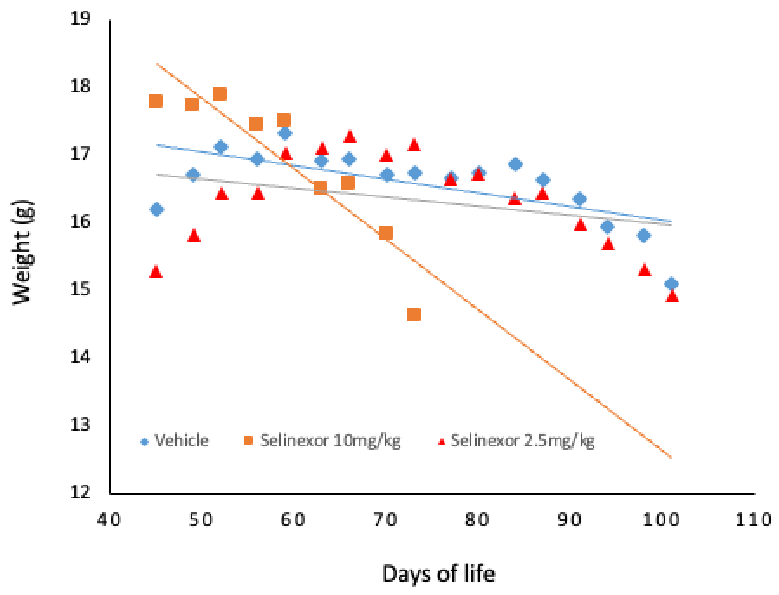

Supplemental Figure 9. Body weight changes in the *Lmna*$^{G609G/G609G}$ **mice over the course of selinexor treatment.** Mean and regression adjustments of weight in the vehicle or selinexor (10 mg/kg or 2.5 mg/kg) treated progeroid mice (n = 10 mice).

Materials and methods

Primary fibroblasts culturing and treatments

Primary human dermal fibroblasts derived from two HGPS patients who are carriers of the classic G608G splice site mutation at the LMNA gene (AG11513, an 8-year-old female donor; and AG11498, a 14-year-old male donor), referred hereafter as HGPS-1 and HGPS-2 cell cultures respectively, and from a healthy subject (AG08469), referred hereafter as a WT cell culture, were acquired from Coriell institute for Medical Research (Camden NJ). Fibroblasts were cultured at

37°C in a humidified 5% CO_2 atmosphere, in Minimal Essential Medium Eagle (MEM; Invitrogen, Carlsbad, CA) supplemented with 15% (v/v) fetal bovine serum (Invitrogen, USA), 100 U/mL penicillin, 10 μg/mL streptomycin and 1 mM sodium pyruvate (Sigma, Saint Louis, MO). When indicated, fibroblasts were treated for 3 or 6 days with 60 nM selinexor (KPT-330, Kariopharm Therapeutics Inc.) diluted in DMSO to a 0.1% final concentration in the culture medium, or for 6 days with 0.15 or 50 nM Leptomycin B (LMB, Sigma) diluted in ethanol to a final concentration of 0.1% in the culture medium. To block autophagy, fibroblasts cultured in Krebs-Ringer-Bicarbonate (KRB) medium (Sigma-Aldrich) were incubated with 50 nM Wortmannin for 6 h, prior to be subjected to WB analysis. All experiments were performed using fibroblast cultures at passage number 8-16.

Cell proliferation assays

Cell viability was determined using the 3-(4,5-dimethylthiazole-2-5-diphenyl tetrazolium bromide kit (MTT; Sigma-Aldrich), according to the manufacturer's recommendations. Briefly, cells cultured on 96-well plates at $1x10^4$ cell density/well were treated for 6 days with the indicated concentrations of selinexor or LMB. Then, the cell samples were washed and resuspended in 100 μL growth medium containing 8 μL MTT (10 mg/mL), prior to be incubated at 37°C with 5% CO_2 for 4 h. Finally, the formazan crystals were dissolved, and the plates was read at 590 nm in an iMarK Microplate Reader (Bio-Rad®).

Senescence-associated β-Galactosidase (SA-β-Gal) assay

Fibroblasts cultured on coverslips were stained with the Senescent Cell Histochemical Staining Kit (Sigma-Aldrich) following the manufacturer's recommendations. Blue-stained cells (senescent cells with higher SA-β-Gal activity) were observed and scored using a light-field inverted microscope (Nikon).

Antibodies

The following primary antibodies were used: Rabbit polyclonal antibodies against lamin A (ab26300), B23 (sc-6013-a), cyclin B1 (sc-752) (Santa Cruz Biotechnology, CA, USA); lamin B1 (ab16048), H3K9me3 (ab8898) (Abcam, Cambridge, UK); LC3 (MBL International, Woburn, MA [PM036]); and CRM1 (Novus Biologicals, CO, USA [NB100-79802]). Mouse monoclonal antibodies against lamin A/C (Abcam, Cambridge, UK [ab8984]), progerin (Santa Cruz Biotechnology, CA, USA [sc-81611]), p21 (Cell Signaling Technology, MA, USA [2946]), and β-actin (a gift from Dr. Manuel Hernández, CINVESTAV, Mexico). P16.

Indirect Immunofluorescence and confocal microscope analysis

Cells seeded on coverslips were fixed with 4% paraformaldehyde for 10 min in PBS, permeabilized, and blocked for 10 min at room temperature with immunofluorescence (IF) buffer (0.1% Triton X-100, 0.02% SDS, and 10 mg/ml BSA), and then incubated overnight at 4°C with the corresponding primary antibodies. The following day, cells were washed with 0.1% Triton X-100 in PBS for 5 min and then with PBS alone, prior to being incubated for 1 h at room temperature with the corresponding fluorochrome-conjugated secondary antibodies (Jackson ImmunoResearch Laboratories). Where indicated F-actin was labeled using TRITC-conjugated Phalloidin (Sigma-Aldrich) diluted 1:150 in IF buffer for 15 min at room temperature. Then, cells were incubated for 15 min at room temperature with 0.2 μg/ml diamino-2-phenylindole (DAPI; Sigma-Aldrich) in PBS for nuclei labeling. After washing, coverslips were mounted on microscope slides with VectaShield (Vector Laboratories, Inc. Burlingame, CA, USA) and examined on a confocal laser scanning microscope (CLSM; Eclipse Ti-E inverted confocal laser scanning microscope (NiKon, Japan). Analysis of digitized images were performed using ImageJ 1.49 software

(http://imageJ.nih.gov.ij). Morphometric analysis of nuclei was performed by determining nuclear area and roundness using Image J 1.46j software. The nucleolar area of > 1000 nucleoli was quantified in maxima projection using a plugin 3D objects counter, and measuring the cross-sectional area occupied by B23 immunostaining with ImageJ software. Quantitative analysis to determine the nuclear to cytoplasmic ratio of fluorescence (Fn/c) was carried out using ImageJ software. To quantify H3K9me3 fluorescent foci, the ImageJ Find Maxima function from was applied. Data were plotted using GraphPad Prism 8 version software.

Live imaging of mitochondria

To assess mitochondrial morphology, fibroblasts cultured on 35-mm glass-bottom dishes at 70% confluence were incubated with 100 nM MitoTracker Green (Cat: M7514, Thermo Fisher Scientific, USA) for 25 minutes. Subsequently, the cells were washed twice with PBS and further stained for 10 minutes with 1 µg/ml Hoechst (Sigma Aldrich; Cat: B2261, USA). The cells were then washed twice with PBS and maintained in fresh medium at 37°C under humidified conditions with 5% CO_2 during imaging. Live imaging was conducted using the Eclipse Ti-E inverted confocal laser scanning microscope (Nikon, Japan), equipped with a Tokai Hit INU on-stage incubator (Spectra Services, Ontario, NY, USA) and a C90 camera. Morphometric analysis of mitochondria was performed using Image J 1.46j software, with a plugin, to determine Feret's diameter (which is the longest distance between any two points along the selection boundary).

Determination of reactive oxygen species

The intracellular levels of reactive oxygen species (ROS) were quantified using the Cellular ROS Assay Kit (Abcam; Cat: ab113851), following the instructions provided by the manufacturer. In brief, fibroblasts were plated on 35-mm glass-bottom dishes until they reached 70% confluence. They were then stained with 40 µM 2'-7'-dichlorodihydrofluorescein-diacetate (DCFH-DA, diluted in culture medium) in the dark for 45 minutes at 37°C with 5% CO_2. The cells were counterstained for 15 minutes with Hoechst to visualize

the nuclei, as previously described. They were then washed twice with PBS and subjected to imaging using a confocal microscope. The green-stained live cells correspond to those positive for reactive oxygen species. The intracellular fluorescence intensity was measured using Image J software, and the data were plotted using GraphPad Prism 8.

Western Blotting

Fibroblast lysates were subjected to electrophoresis on 12% SDS-polyacrylamide gels and transferred onto nitrocellulose membranes (Bio-Rad Laboratories, Hercules CA, USA) using a semi-dry transblot apparatus (Bio-Rad Laboratories) for 1 h. The membranes were blocked in TBST [100 mM Tris-HCl pH 8.0, 150 mM NaCl, 0.5% (v/v) Tween-20] with 5% low-fat dried milk and incubated at 4ºC overnight with the proper primary antibodies and the protein signal was developed using horseradish peroxidase-conjugate secondary antibodies (Sigma-Aldrich, MO, USA) and the enhanced chemiluminescence Western blotting detection system (ECL™; Amersham Pharmacia, GE Healthcare), according to the manufacturer´s instructions. Blot images were acquired using C-Digit® Blot Scanner (LI-COR Biosciences, Lincoln, Nebraska USA). For the analysis of mouse tissues, 20 mg of tissue was homogenized in RIPA lysis buffer with a cold protease inhibitor cocktail (Roche complete™, Sigma-Aldrich Inc, St. Louis, MO). The resulting supernatant was stored at -70°C. The protein concentration was determined by the bicinchoninic acid method, as described by the manufacturer (Thermo Fisher Scientific, Inc., Waltham, MA). Subsequently, 50 μg of total protein per sample in laemmli buffer was loaded for electrophoresis into 4% to 15% precast gels (Mini-PROTEAN® TGX™ Precast Protein Gels, BIO-RAD, Hercules, CA). The proteins were electrophoretically transferred onto PVDF membranes (Sigma-Aldrich Inc, St. Louis, MO), and incubated for 24 hours at 4°C with the appropriate primary antibodies. The, the membranes were incubated with secondary antibodies coupled with fluorochromes and analyzed with an Oddissey CLx imaging system (LI-COR Biosciences, Lincoln, NE).

Annexin V assays

Apoptosis was determined using flow cytometry and a commercial Annexin-V Kit (Muse®, Luminex; Austin, Texas, USA), following the manufacturer's protocol. Briefly, attached cells were harvested and centrifuged at 1,400 rpm for 5 min, and the pellet was resuspended in 100 μL growth medium adding 100 μL Annexin V kit to each sample and further incubated for 15 min, prior to be read in the cell analyzer Muse® (Luminex; Austin, Texas, USA). In parallel experiments, fibroblasts were treated for 12 h with 2 μM staurosporine (Sigma-Aldrich; Merck KGaA, Darmstadt, Germany) to induce apoptosis (control).

Cell cycle analysis by flow cytometry

Fibroblasts seeded on 60 mm cell tissues were centrifuged and the pellets was resuspended in 80% cold ethanol for 30 min at 4°C. Samples were then stained in darkness with 1 μg/ml DAPI (Sigma-Aldrich) for 30 min at room temperature. After that, cell samples were transferred to flow cytometer tubes for cell cycle analysis using BD LSR-Fortessa flow cytometer (BD Biosciences, San Jose, CA, USA), and ModFit LT software (Verity Software House, Topsham, ME). Data were plotted using GraphPad Prism 8 software.

Cell viability assay

Cell viability was analyzed using the MTT (3-(4,5-dimethylthiazol-2-yl)-2,5-diphenyltetrazolium bromide) kit, following the manufacturer's instructions (Sigma-Aldrich). Briefly, cells were cultured for 24 h, prior to be treated for 24 or 72 h with the indicated concentrations of selinexor or LMB. Afterwards, the supernatant was discarded, and the cells were washed twice with PBS 1X and added with 100 μL of growth medium containing 8 μL MTT (10 mg/mL) prior to be incubated at 37°C with 5% CO_2 for 4 h. Finally, the crystals were dissolved, and the plate was read at 590 nm in an iMarK Microplate Reader (Bio-Rad®).

Reverse transcription-quantitative polymerase chain reaction (RT-qPCR)

Total RNA was extracted from fibroblasts using TRIzol Reagent (Invitrogen, Carlsbad, CA, USA), according to the manufacturer's instructions. RNA purity and concentration were determined using a NanoDrop 2000 spectrophotometer (NanoDrop Technologies, Wilmington, DE, USA). Aliquot of RNA (1 µg) were used to prepare cDNA with the High-Capacity cDNA reverse transcription kit (Thermo Fisher Scientific. Waltham, MA, USA), following the manufacturer's protocol. Quantitative PCR reactions were carried out in 20 µL using TaqMan probe-based assays and following the provider´s instructions. Specific primers for progerin detection(Harhouri et al., 2017) and its corresponding Taqman probe (CGCTGAGTACAACCT; Applied Biosystems, Foster City, CA, USA), as well as predesigned primers for the endogenous control PolR2A (Hs00172187_m1) were employed. The relative progerin mRNA expression was calculated by the $2^{\Delta\Delta CT}$ method.

RNA sequencing

Total RNA was extracted using TRIZOL (Invitrogen, Gaithersburg, MD, USA) and was then treated with RNase-free DnaseI to remove the genomic DNA, in which DnaseI was inactivated in the presence of EDTA for incubation at 65 °C according to manufacturer's protocol. The RNA integrity was assessed using the Bioanalyzer 2100 system (Agilent Technologies, Santa Clara, CA, USA). The RNA purity was checked using the NanoPhotometer spectrophotometer (IMPLEN, Westlake Village, CA, USA) and the RNA concentration was measured using the Qubit RNA Assay Kit in Qubit2.0 Fluorometer (Life Technologies). We generated strand-specific RNA-seq libraries (using the Illumina TruSeq Stranded mRNA Library protocol) for a total of 16 samples for four different experimental conditions: vehicle-treated WT cells: vehicle-treated HGPS-1 cells;

selinexor-treated WT cells and selinexor-treated HGPS-1 cells. Each condition included four biological replicates. RNA-seq libraries were sequenced in the Unidad Universitaria de Secuenciación Masiva of the University of Mexico. We sequenced an average of 22 million 75 bp paired-end reads per library (+- 8 million reads). Reads were trimmed using trim_galore (https://github.com/FelixKrueger/TrimGalore, v.1.16). We downloaded the human genome from the ENSEMBL database (https://www.ensembl.org/index.html; v.107) and then mapped the reads using Hisat2 (D. Kim, Langmead, & Salzberg, 2015). We calculated read counts per gene using htseq-count (v0.9.1) (Putri, Anders, Pyl, Pimanda, & Zanini, 2022). Read counts for the 16 samples were loaded into the R package (https://www.r-project.org/) as a data frame. We used the EdgeR package (Robinson, McCarthy, & Smyth, 2010) to perform differential expression analyses of genes between the WT and the other conditions (normalization using TMM -Trimmed Mean of M-values-, FDR -False Discovery Rate- set at 0.05). All statistical analyses were performed using the R package, standard libraries. Data were plotted using the R package, "ggplot2" library (https://ggplot2.tidyverse.org/). Data generated for this project are available from the NCBI database (http://www.ncbi.nlm.nih.gov/) under Bioproject PRJNA1129851.

Mice

The transgenic Lmna$^{G609G/G609G}$ mouse strain (Osorio et al., 2011) and its corresponding wild-type littermate (C57BL/6J), provided by Carlos López-Otín (Universidad de Oviedo, Asturias, Oviedo, Spain), were fed ad libitum with regular food supplemented with Nutri-Cal® oral gel (Vetoquinol, CA) twice per week. Mice were housed in ventilated cages kept in bio-bubbles (portable clean rooms) and protected by a double HEPA filtration system, in the Production and Experimentation of Laboratory Animals Unit (UPEAL-CINVESTAV, CDMX, Mexico). The experimental

procedures were conducted in concordance with the Institutional Animal Use and Care guideless (Mexican official norm, NOM-062-ZOO-1999), and the corresponding approved protocol (No. 0314-20). Routinely genotyping of progeroid mice was carried out by standard PCR assays as previously(Osorio et al., 2011). *Selinexor treatment*. Unless otherwise stated, male and female Lmna$^{G609G/G609G}$ homozygous mice were administrated by oral gavage with selinexor (2.5mg/kg) diluted in the vehicle (0.6% Polaxamer pluronic F-68 + 0.6% Plasdone PVP K-29/32 in water) or with the vehicle alone (control mice), twice a week from 5 to 14 weeks of age. Mice were euthanized at the experimental endpoint. *Histopathological analysis*. Tissue samples were fixed with 4% paraformaldehyde for two hours and then embedded in paraffin. Sections of liver and aorta (10-25 μm thick) were obtained using microtome and subjected to immunofluorescence analysis using the indicated primary and secondary antibodies. Tissue samples were analyzed by confocal microscopy as above.
